# Retinal and cerebral hemodynamics redistribute to favor thermoregulation in response to passive environmental heating and heated exercise in humans

**DOI:** 10.1080/23328940.2024.2411771

**Published:** 2024-10-16

**Authors:** Harrison T. Caddy, Jesse L. Criddle, Kristanti W. Wigati, Howard H. Carter, Lachlan J. Kelsey, Alla Soloshenko, William H. Morgan, Barry J. Doyle, Daniel J. Green

**Affiliations:** a Vascular Engineering Laboratory, Harry Perkins Institute of Medical Research, Queen Elizabeth II Medical Centre, Nedlands, Australia; b The UWA Centre for Medical Research, The University of Western Australia, Perth, Australia; c School of Human Sciences (Exercise and Sport Sciences), The University of Western Australia, Perth, Australia; d Medical Physiology and Biochemistry Department, Faculty of Medicine, Universitas Airlangga, Surabaya, Indonesia; e School of Engineering, The University of Western Australia, Perth, Australia; f Lions Eye Institute, Perth, Australia; g Centre for Ophthalmology and Visual Science, The University of Western Australia, Perth, Australia; h International Space Centre, Perth, Australia

**Keywords:** Environmental heat, exercise, retina, cerebrovascular, hemodynamics, simulation, computational fluid dynamics

## Abstract

Core temperature (T_C_) changes, alongside exercise, affect hemodynamic responses across different conduit and microvascular beds. This study investigated impacts of ecologically valid environmental heat and exercise exposures on cerebral, skin and retinal vascular responses by combining physiological assessments alongside computational fluid dynamics (CFD) modeling. Young, healthy participants (*n* = 12) were exposed to environmental passive heating (PH), and heated exercise (HE) (ergometer cycling), in climate-controlled conditions (50 mins, 40°C, 50% relative humidity) while maintaining upright posture. Blood flow responses in the common carotid (CCA), internal carotid (ICA) and central retinal (CRA) arteries were assessed using Duplex ultrasound, while forearm skin microvascular blood flow responses were measured using optical coherence tomography angiography. Three-dimensional retinal hemodynamics (flow and pressure) were calculated via CFD simulation, enabling assessment of wall shear stress (WSS). T_C_ rose following PH (+0.2°C, *p* = 0.004) and HE (+1.4°C, *p* < 0.001). PH increased skin microvascular blood flow (*p* < 0.001), whereas microvascular CRA flow decreased (*p* = 0.038), despite unchanged ICA flow. HE exacerbated these differences, with increased CCA flow (*p* = 0.007), unchanging ICA flow and decreased CRA flow (*p* < 0.001), and interactions between vascular (CCA vs. ICA *p* = 0.018; CCA vs. CRA *p* = 0.004) and microvascular (skin vs. retinal arteriolar *p* < 0.001) territories. Simulations revealed patterns of WSS and lumen pressure that uniformly decreased following HE. Under ecologically valid thermal challenge, different responses occur in distinct conduit and microvascular territories, with blood flow distribution favoring systemic thermoregulation, while flow may redistribute within the brain.

## Introduction

Humans are commonly exposed to a variety of thermal stressors stemming from exposure to heated environments and/or physical activity, with different tolerability, thermoregulatory and cardiovascular responses observed [1,2]. Effective thermoregulation in humans is influenced by changes in cardiac output [3] alongside cardiovascular reflex-mediated redistribution of blood flow from inactive regions to those responsible for heat loss [4]. In response to passive thermal loads, this is achieved via skin microvascular vasodilation in the context of increased heart rate (HR) and cardiac output [5]. The imposition of endogenous exercise on an underlying heat load (i.e. exercise in the heat) represents a significant challenge to cardiovascular homeostasis [6], as competition arises between increasing blood flow to the muscle to subserve energy demands, blood flow to the skin microvasculature to effect heat loss (in combination with sweat production), and blood flow to the brain to maintain consciousness [5,7]. Exercise in the heat is therefore associated with integrative physiological responses and large redistributions of blood flow, including to microvessels in the skin, the brain and by extension, the eye.

The retinal vasculature [8] has been proposed as a surrogate for cerebral microvascular function [9] and generalized microvasculature health in humans [10,11]. It can also be adversely affected by hemodynamics in proximal cerebral feed arteries such as the internal carotid artery (ICA) [12]. Understanding the effects of different thermal stressors on retinal hemodynamics alongside conduit feed arteries and other microvascular beds may provide insight into the contributory factors associated with ecologically valid environmental exposures. Combining retinal artery ultrasound measures [13,14] with subject-specific 3D computational fluid dynamics (CFD) simulation further enables quantitative assessment of retinal microvascular responses, and by association, an indication of cerebral microvascular hemodynamics in response to environmental heat and/or exercise exposure.

No previous study has combined direct extracranial and retinal hemodynamic assessments, alongside systemic thermoregulatory and cardiovascular measures, during environmental heating and/or exercise in humans. This study aimed to compare changes in novel microvascular assessments of skin and retina blood flow, alongside extracranial and microvascular hemodynamic changes, in response to passive heat and heated exercise exposure. This incorporated development of a simulation framework to calculate changes in 3D hemodynamics in retinal vessels. Our rationale for comparing environmental passive heating and heated exercise across identical time-periods was based on ecological validity; we sought to compare regional blood flow and hemodynamic changes under circumstances similar to those commonly experienced by humans exposed to hot environments, in which moderate exercise may also be undertaken. We hypothesized that hemodynamic measures (e.g. blood velocity and flow, shear stress) in the common carotid (CCA), ICA and retinal arteries, alongside skin microvasculature, would increase in response to environmental heating and/or heating during exercise, and that exercise in the heat would induce larger changes than heating alone.

## Materials and methods

### Physiological testing

The experimental methods used in this study were approved by the University of Western Australia (UWA) Human Research Ethics Committee (2021/ET00005). Young healthy participants (*n* = 12; six males, six females, age: 25.9 ± 3.6 years, BMI: 22.5 ± 2.2 kg·m^−2^) were recruited locally from residents in Perth, Western Australia, following initial consultation, application of exclusion criteria and provision of written informed consent. Of the 6 female participants, 4 were actively taking oral contraceptives, while the remaining 2 were tested within the follicular and luteal menstrual cycle phases, respectively. Exclusion criteria were as follows: adverse cardiovascular history (e.g. myocardial infarction, stroke, diabetes type 1 or 2), or implanted devices (e.g. pacemaker), age (>18; <60 years), hypertension (resting systolic >160 mm Hg or diastolic >100 mm Hg), smoking status, high BMI (>35 kg·m^−2^), low body weight (<37 kg), and any histories of obstructive gastro-intestinal disease/surgery, ocular disorders/surgeries (e.g. glaucoma, refractive eye surgery) and corneal conditions (e.g. epithelial erosion).

#### Baseline VO_2_peak testing

Participants underwent an initial VO_2_peak test involving expired gas analysis (TrueOne 2400, Parvo Medics, Salt Lake City, United States) on a stationary cycle ergometer (Excalibur Sport, Lode BV, Groningen, the Netherlands). This test consisted of a 3-min warm-up phase cycling at 50W, followed by 25W increments in pedal resistance per min until voluntary termination. Participants were instructed to maintain a cadence between 50 and 70 rpm. HR was measured continuously (Polar H10, Polar, Kempele, Finland) throughout the test to obtain a cycling peak HR.

#### Experimental procedure

Following the test above, participants underwent two separate experimental sessions, sitting passively under environmental heating with minimal exertion (i.e. PH), or exercising under the same conditions in the heat (i.e. HE). Prior to each visit, participants were instructed to refrain from consuming any caffeine and alcohol, as well as undertaking vigorous exercise within the 24 h prior to each visit [15]. Baseline measures were collected under ambient conditions which were consistent on each day (23°C, 45% relative humidity (RH), wet bulb temperature (WBT) = 15.5°C). To mitigate influences of changes in posture, an upright posture was maintained throughout all tests and imaging protocols.

For the PH session, participants remained seated in an upright position in a chair while placed in a climate-controlled chamber for 50 mins at 40°C, 50% RH, WBT = 30.9°C. On a separate day, and separated by at least 48 h, participants underwent the HE session, cycling continuously on an ergometer (Ergomedic 828 E, MONARK, Vansbro, Sweden) for 50 mins in the same environmental conditions (40°C, 50% RH, WBT = 30.9°C). Exercise intensity was set to 45% VO_2_peak to emulate typical moderate exercise (e.g. in a vocational context), and to maintain consistency with previous studies of combined endogenous and environmental heating [1,2,16]. This was reduced in 5W increments at 5-min bands of testing as required to maintain HR at or below 80% of peak HR (obtained during the VO_2_peak test). Across both visits while in the chamber, participants were provided *ad libitum* access to thermoneutral water and seated upright posture was maintained. Testing was terminated early if T_C_ rose above 39°C.

#### Experimental measures

Unless otherwise stated (below), all experimental measures were assessed under baseline conditions (23°C, 45% RH, WBT = 15.5°C) and immediately following 50 mins of either PH exposure (40°C, 50% RH, WBT = 30.9°C) or HE (40°C, 50% RH, WBT = 30.9°C), while participants remained inside the climate chamber.

#### Core temperature

Core temperature (T_C_) was measured using a wireless temperature capsule (eCelcius Performance Capsule, BodyCap, Hérouville Saint-Clair, France), ingested approximately 5–12 h prior to each testing session [17].

#### Corneal surface thermal imaging

Corneal surface temperature was measured using an infrared thermal imaging smartphone attachment (CompactPRO, Seek Thermal, Santa Barbara, United States). To assist with camera focus, a 3D printed macro lens attachment was also used. From this image, corneal surface temperature was estimated using a custom MATLAB (v2019b, Mathworks, Natick, United States) script.

#### Systemic hemodynamics, energetics and pressures

Mean arterial pressure (MAP) was measured manually using a stethoscope and pneumatic arm cuff (FlexiPort 11 & DuraShock DS66, Welch Allyn, Skaneateles Falls, United States). Intraocular pressure (IOP) within the eye was measured using a handheld rebound tonometer (TA01i, iCare, Vantaa, Finland). Ocular perfusion pressure (OPP) was calculated as 2/3 MAP – IOP, which is consistent with a seated upright position [18]. HR was continuously measured using a wearable HR sensor (Polar H10) and peripheral oxygen saturation (O_2_) was measured using a handheld pulse oximeter (PC-66 h, CMI Health, Alpharetta, United States). Expired oxygen (VO_2_) and mixed expired partial pressure of carbon dioxide (PeCO_2_) were measured at rest prior to entering the chamber, and then again at the mid and endpoints of chamber exposure (TrueOne 2400).

#### Ultrasound assessments of retinal and extracranial artery blood flows

Blood flow was measured in the left CCA and ICA using high-resolution Duplex ultrasound methods [19–23]. Blood flow velocity (BFv) measured using Doppler velocity was captured simultaneously with lumen diameter (B-mode imaging) using a linear transducer (15L4A) and portable ultrasound system (uSmart 3300, Terason, Burlington, United States). Arterial lumen edge detection and wall tracking software, combined with velocity envelope capture, were used to calculate blood flow (BF) in these arteries, as previously described [24,25].

BFv within the left central retinal artery (CRA) was measured using a linear array transducer with Doppler velocity and color Doppler imaging (15L4, uSmart 3300), using standardized methods [13]. Sterile ultrasound gel was used to couple the probe with the closed eyelid. To ensure safe insonation, ophthalmic probe settings were used which limited thermal and mechanical indices, as per existing guidelines and recommendations (thermal index < 1; mechanical index < 0.3) [26]. BF in the CRA was then calculated by combining velocity measures with estimated CRA diameter (see Structural Eye Imaging section in the Supplementary Information which describes CRA diameter estimation in further detail).

All ultrasound scans were performed in an upright seated position, and within the climate chamber where applicable (i.e. post PH or HE). To minimize variability, sonographers and devices remained consistent across both visits for each participant [15,27–29].

#### Skin blood flow

Optical coherence tomography angiography (OCTA) was used to non-invasively image the skin microvasculature, as detailed in previous studies [30,31]. A 5 mm square patch of skin was selected on the ventral side of the forearm, with a thin layer of brine solution applied to the skin prior to lowering the optical coherence tomography (OCT) probe, which was fixed in place for the duration of the scan. The OCT imaging scanner (Telesto II, ThorLabs, Newton, United States) acquired a 3D scan of the skin subsurface microvasculature, which was focused to a depth of 300 µm [30]. Scans were acquired over a 2-min period at a frequency of 76 kHz. Imaging analysis scripts previously developed in MATLAB (v2019b) [30,31] were then used to quantify median diameter, blood velocity and flow in the micro vessels in forearm skin. The OCTA probe contact location on the forearm was marked to maintain spatial location between tests and visits.

Independently, forearm skin red blood cell flux was also measured using laser Doppler flowmetry (LDF) (Periflux 5000 System, Perimed, Järfälla, Sweden) to obtain an index of general skin blood flux [30,31]. The Doppler probe (Model 413, Perimed) was fixed to the ventral side of forearm using double-sided adhesive adjacent to the OCTA scan location. Data was collected simultaneously with the OCTA scan using LabChart 7 (AD Instruments, Sydney, Australia), and averaged over the same period (2 mins).

#### Questionnaire

Following PH or HE, each participant completed a version of the environmental symptoms questionnaire [32,33] specifically modified for heat stress symptom assessment. The questionnaire consisted of a 6-point scale response system and contained 22 questions relating to heat stress-related symptoms (e.g. lightheadedness, sweatiness, thirst).

### Retinal artery plexus simulation

To derive 3D localized hemodynamics within participant retinal vessels (e.g. wall shear stress (WSS), lumen pressure, microvascular outlet flows), structural imaging of the left eye, 3D model construction and CFD simulation was performed.

#### Three-dimensional model construction

**Figure 1.**
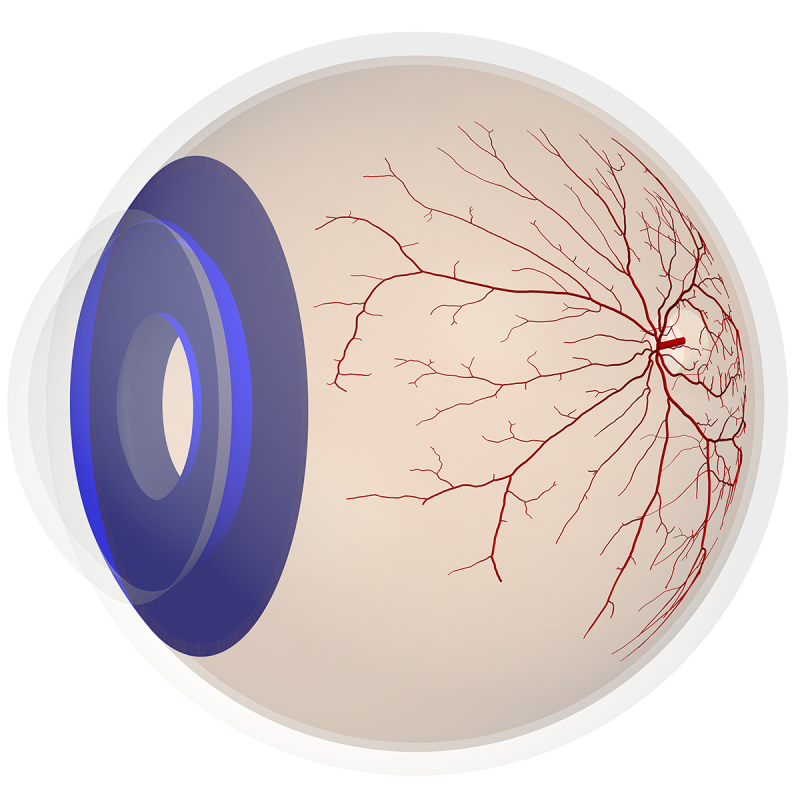
An example of a reconstructed three-dimensional (3D) eye model with embedded retinal artery plexus. The lofted centerline and vessel diameter data from ultra-widefield fundus (UWF) imaging was projected to the retinal spherical curvature radius calculated from a parametric computer aided design (CAD) model using measured input data from structural eye imaging.

To obtain the retinal spherical curvature radius, a parametric computer aided design (CAD) model of the eye was generated for each participant using input data from structural eye imaging. This imaging consisted of Scheimpflug imaging, ocular ultrasound and retinal OCT. Retinal arteriolar vessel centerline and diameter information were extracted from ultra-widefield fundus (UWF) images and projected to the calculated retinal spherical curvature radius. The diameter of the CRA was estimated using the central retinal artery equivalent (CRAE) from a fundus image extracted from OCT imaging [34–37] (see Structural Eye Imaging section in the Supplementary Information, which explains these imaging modalities as per Supplementary Figure S1 and 3D reconstruction techniques as per Supplementary Figure S2 in further detail). An example of a reconstructed eye displaying the projected 3D retinal artery plexus is presented in Figure 1.

#### Computational fluid dynamics

Localized 3D vessel hemodynamics within the retina for each participant were calculated from solving the Navier–Stokes equations using CFD simulations developed in STAR-CCM+ (v18.02.010, Siemens, Munich, Germany), utilizing measured data from two experimental conditions; at resting baseline (i.e. R_HE_) and following HE (see Supplementary Figure S3, which presents inlet flow waveforms for each case from R_HE_ and HE conditions respectively). Due to computational limitations, simulations were conducted for R_HE_ and HE only. These conditions were selected as HE was postulated as the greater stressor of the two conditions (i.e. compared to PH). The simulation methods extend on previous work [38,39]. Briefly, blood was assumed to be incompressible and viscosity was modeled to account for the Fåhræus–Lindqvist effect, whilst the lumen was assumed to be rigid and 3-element Windkessel models (with minimum pressure limited by IOP measurement) were employed at each of the arteriole outlets (see the Computational Fluid Dynamics Simulations section of the Supplementary Information, which provides further details regarding computational meshing, mesh independence studies, model assumptions, boundary conditions and computational execution – Supplementary Tables 1–3). From these simulations, we calculated instantaneous WSS, lumen wall pressure and flow at the retinal arteriole outlet surfaces, which were then averaged over a cardiac cycle to obtain time-averaged data.

### Data collection and analysis

Skewness and Shapiro–Wilk testing was used to ascertain normality. Within variable responses were investigated using 2-way repeated measures ANOVA, with post-hoc analysis conducted using paired *t*-tests. Responses between arteries were assessed using 2-way repeated measures MANOVA with subsequent Hotelling T-square analysis. P-values <0.05 were deemed statistically significant. Where applicable, data are presented as group mean ± standard deviation.

## Results

The peak HR recorded during the preliminary cycle exercise test was 178 ± 8 bpm and VO_2_peak was 44.4 ± 11.2 mL·kg^−1^·min^−1^. Twelve subjects completed 50 mins of chamber exposure under the PH condition. In response to HE, one participant exhibited a T_C_ rise above 39°C after 35 mins; the exercise testing was terminated at this time and measures were collected whilst the participant remained in the climate chamber. Summary data for the HE condition (*n* = 12) include this participant despite the final measure being collected 15 mins prematurely. Outside of this case, all responses presented refer to time points either at rest (prior to exposure to either PH or HE stimuli) and immediately following exposure (i.e. ~50 minutes of PH and HE, respectively).

### Cardiovascular responses to passive heat and heated exercise

**Figure 2.**
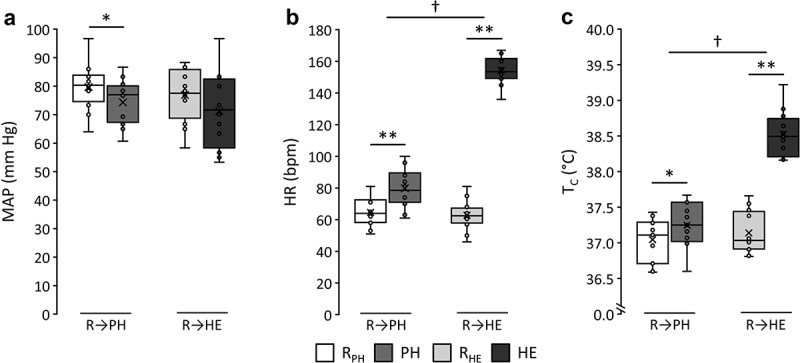
Mean arterial pressure (MAP) (a), heart rate (HR) (b) and core body temperature (T_C_) (c) responses to environmental passive heating (PH) from rest (R_PH_) and to heated exercise (HE) from rest (R_HE_). Stars (*) indicate the level of significance for a main effect of time (*p < 0.05; **p < 0.001) using paired *t*-tests while crosses (†) indicate significant interactions (†*p* < 0.05; ††*p* < 0.001).

MAP, HR and T_C_ responses to PH and HE are presented in Figure 2, and where additional cardiorespiratory (i.e. VO_2_, PeCO_2_, O_2_ saturation) and ocular (i.e. IOP, OPP and corneal temperature) responses were collected (Supplementary Figure S4). Heat stress symptom score responses to PH and HE were also collected (Supplementary Figure S5).

For MAP, 2-way ANOVA revealed a main effect of time (*p* = 0.026), indicating that the interventions were associated with a decrease in MAP compared to resting measures. Whilst no significant interaction was apparent, post-hoc paired *t*-tests indicated that MAP was significantly lower after PH compared to baseline (74 ± 8 vs. 80 ± 8 mm Hg, *p* = 0.036), whereas the decrease in response to HE did not achieve statistical significance (71 ± 13 vs. 77 ± 9 mm Hg, *p* = 0.109).

For HR, a main effect was found for time (*p* < 0.001), as well as an interaction (*p* < 0.001). Post-hoc testing revealed that, compared to baseline, HR was significantly higher following both PH (80 ± 12 vs. 65 ± 9 bpm, *p* < 0.001) and HE (155 ± 9 vs. 63 ± 10 bpm, *p* < 0.001). HE had a significantly larger impact on HR than PH (*p* < 0.001).

T_C_ during PH in one case was unable to be collected – this case was excluded from comparing interactions for this variable. Similar to HR, T_C_ revealed a main effect across time (*p* < 0.001) and a significant interaction between conditions (*p* < 0.001). Post-hoc analysis showed both PH (37.2 ± 0.3 vs. 37.0 ± 0.3°C, *p* = 0.004) and HE (38.5 ± 0.3 vs. 37.1 ± 0.3°C, *p* < 0.001) increased T_C_ compared to their respective baselines, and that this effect was significantly larger following HE than PH (*p* < 0.001).

### Arterial responses to passive heat and heated exercise

The ICA in one case was unable to be insonated due to anatomical limitations – this case was excluded from arterial response comparison.

#### Comparison within arteries between passive heat and heated exercise conditions

**Figure 3.**
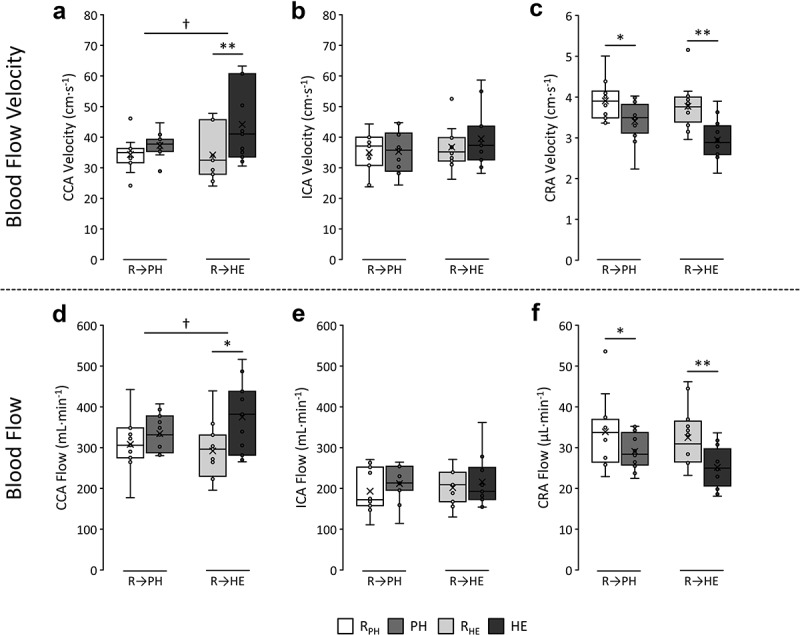
Common carotid (CCA) (a), internal carotid (ICA) (b), and central retinal (CRA) (c) artery blood flow velocity responses, and CCA (d), ICA (e) and CRA (f) blood flow responses to environmental passive heating (PH) from rest (R_PH_) and heated exercise (HE) from rest (R_HE_). Stars (*) indicate the level of significance for a main effect of time (*p < 0.05; **p < 0.001) using paired *t*-tests while crosses (†) indicate significant interactions (†*p* < 0.05; ††*p* < 0.001).

Time averaged BFv and BF responses within the left CCA, ICA and CRA in response to PH and HE are presented in Figure 3.

For the CCA, 2-way ANOVA revealed a main effect of time for both BFv (*p* < 0.001) and BF (*p* = 0.012), and also an interaction for both variables (BFv *p* = 0.025; BF *p* = 0.032). Post-hoc paired *t*-tests indicated that while BFv and BF generally increased in response to both PH and HE, only responses to HE from baseline significantly increased BFv (44.2 ± 12.5 vs. 34.2 ± 8.5 cm·s^−1^, *p* < 0.001) and BF (375.2 ± 88.7 vs. 292.1 ± 68.5 mL·min^−1^, *p* = 0.007). Furthermore, the BFv (*p* = 0.025) and BF (*p* = 0.032) responses to HE were larger than to PH alone.

In the ICA, no significant main effects, nor interactions for either BFv or BF were observed, indicating responses to PH and HE remained constant in this artery – irrespective of exposure.

In the CRA, a main effect of time was found for both BFv (*p* < 0.001) and BF (*p* = 0.002). Post-hoc paired *t*-tests revealed significant decreases for both BFv (3.4 ± 0.5 vs. 3.9 ± 0.5 cm·s^−1^, *p* = 0.021) and BF (29.1 ± 4.3 vs. 33.9 ± 8.5 µL·min^−1^, *p* = 0.038) in response to PH compared to resting data. Similarly, decreases were also observed for BFv (2.9 ± 0.5 vs. 3.8 ± 0.6 cm·s^−1^, *p* < 0.001) and BF (25.2 ± 5.0 vs. 32.5 ± 7.3 µL·min^−1^, *p* < 0.001) following HE. No significant interaction was present between PH or HE.

#### Comparison between arteries in response to passive heat or heated exercise conditions

**Figure 4.**
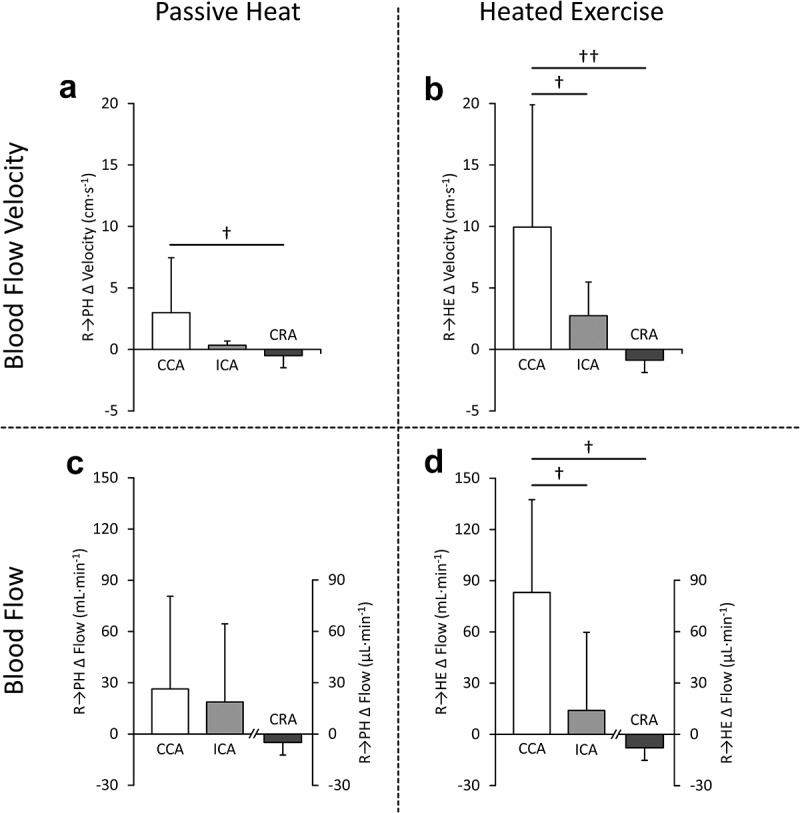
Between artery comparisons of the change (Δ) in velocity in response to either environmental passive heating (PH) from rest (R_PH_) (a) or heated exercise (HE) from rest (R_HE_) (b). Between artery comparisons of changes in flow are also presented in response to PH (c) or HE (d) from rest. The common carotid artery (CCA), internal carotid artery (ICA) and central retinal artery (CRA) changes in velocity or flow are shown in each plot. For change in flow measures, CRA data is presented using a secondary vertical axis. Crosses (†) indicate a significant interaction in responses between arteries on underlying absolute velocity or flow data (†*p* < 0.05; ††*p* < 0.001).

To aid in visualization of interactions between arteries, changes in BFv and BF responses to either PH or HE from respective baseline measures between the ICA, CCA and CRA are presented in Figure 4.

Using 2-way repeated measures MANOVA, no interaction between the CCA and ICA in the magnitude of change for either BFv or BF in response to PH was found. Conversely, in response to HE, CCA BFv (interaction *p* = 0.043) and BF (interaction *p* = 0.018) increased significantly more than the changes observed in the ICA.

When ICA and CRA changes were compared, the small increases in ICA BFv and BF in response to both PH and HE were not significantly different from the decreases observed in the CRA (i.e. the interactions were not significant).

Comparisons between the changes (from baseline) in CCA and CRA responses to PH revealed a significant interaction for BFv (*p* = 0.030) only; CCA increased, whereas CRA decreased. In response to HE, changes in BFv (interaction *p* < 0.001) and BF (interaction *p* = 0.004) were found, indicating an inverse response between arteries with CCA measures increasing while CRA measures decreased.

### Microvascular responses to passive heat and/or heated exercise

Eye geometry measurements for each eye component, as well as cohort geometry statistics from the 3D retinal arterioles utilized in CFD simulations were calculated (see Supplementary Figure S6 and Supplementary Tables 4–6). Visualizations of surface mapped 3D vessel diameter distributions were also plotted for each case (see Supplementary Figure S7).

#### Comparisons of skin and retinal microvasculature

**Figure 5.**
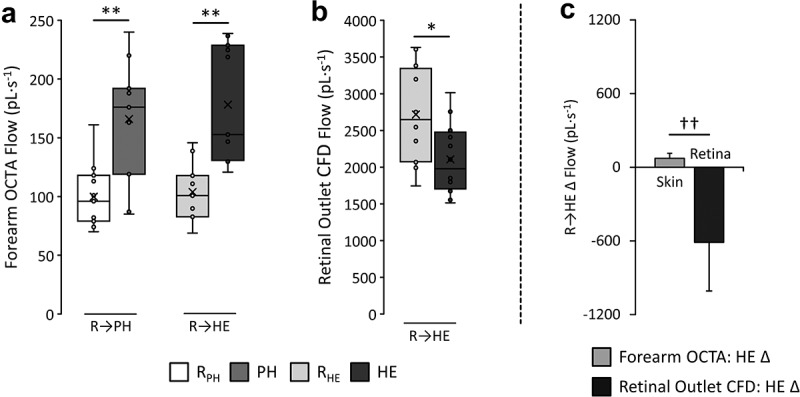
Skin blood flow measurements using optical coherence tomography angiography (OCTA) in the forearm (a) in response to environmental passive heating (PH) from rest (R_PH_) and heated exercise (HE) from rest (R_HE_). Retinal arteriole outlet blood flow responses to HE from R_HE_, which were calculated using computational fluid dynamics (CFD) simulation, are also shown (b). Between arteriolar microvascular network comparisons of the change (Δ) in microvascular flow in response to HE from rest are presented (c). Stars (*) indicate the level of significance between variables (*p < 0.05; **p < 0.001) using paired *t*-tests. Crosses (†) indicate a significant interaction in arterial microvasculature responses (†*p* < 0.05; ††*p* < 0.001).

Skin OCTA scans collected during PH in one case were unable to be collected – this case was excluded from comparing interactions. Microvasculature flow responses to either PH and/or HE can be found in Figure 5. For forearm OCTA flow, 2-way ANOVA revealed a main effect for time (*p* < 0.001), but no significant interaction was present. Post-hoc paired *t*-tests found skin microvasculature flow was significantly higher in response to PH from rest (166.0 ± 50.4 vs. 99.8 ± 27.5 pL·s^−1^, *p* < 0.001). Similarly, flow was significantly higher in response to HE compared to rest (178.4 ± 50.2 vs. 104.1 ± 24.0 pL·s^−1^, *p* < 0.001).

Conversely, using paired *t*-tests, average BF across retinal outlets calculated from CFD (Figure 5b) significantly decreased in response to HE compared to rest (2105.6 ± 488.1 vs. 2716.6 ± 674.8 pL·s^−1^, *p* < 0.001).

To aid in visualization of microvascular network interactions, changes in flow responses specifically to HE from rest between forearm OCTA data and calculated CFD flow at the retinal outlets are presented in Figure 5c. Comparing between microvasculature responses, 2-way repeated measures MANOVA revealed a significant interaction between flow in forearm skin and at the retinal outlets (*p* < 0.001), indicating that as skin BF increases, retinal microvascular flow decreases in response to HE.

Skin blood flow flux measured using LDF were also recorded (Supplementary Figure 8).

#### Localized retinal hemodynamics in response to heated exercise

**Figure 6.**
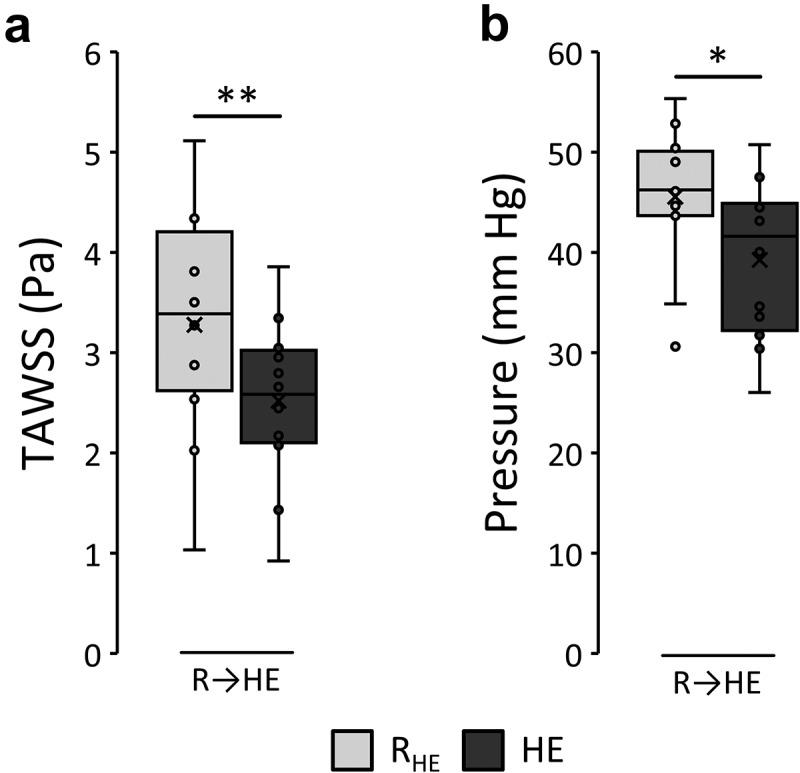
Computational fluid dynamics (CFD) calculated surface average responses from rest (R_HE_) to heated exercise (HE) for time-averaged wall shear stress (TAWSS) (a) and lumen wall pressure (b). Stars (*) indicate the level of significance between variables (*p < 0.05; **p < 0.001) using paired *t*-tests.

3D surface averaged and cardiac cycle time-averaged wall shear stress (TAWSS) and lumen pressure responses at baseline and following HE calculated using CFD simulation are presented in Figure 6. In response to HE, paired *t*-tests revealed TAWSS significantly decreased (2.52 ± 0.80 vs. 3.28 ± 1.11 Pa, *p* < 0.001). Similarly, time-averaged lumen pressure was found to be significantly lower in response to HE (39.28 ± 7.75 vs. 45.58 ± 7.03 mm Hg, *p* = 0.027). 3D vessel distributions of TAWSS and pressure (see Supplementary Figure 9, which depicts absolute TAWSS and pressure in an example case), as well as the relative changes in these metrics (see Supplementary Figure 10, which shows the relative change in TAWSS and pressure in response to HE across all cases) revealed qualitative decreases across cases in response to HE compared to rest.

**Figure 7.**
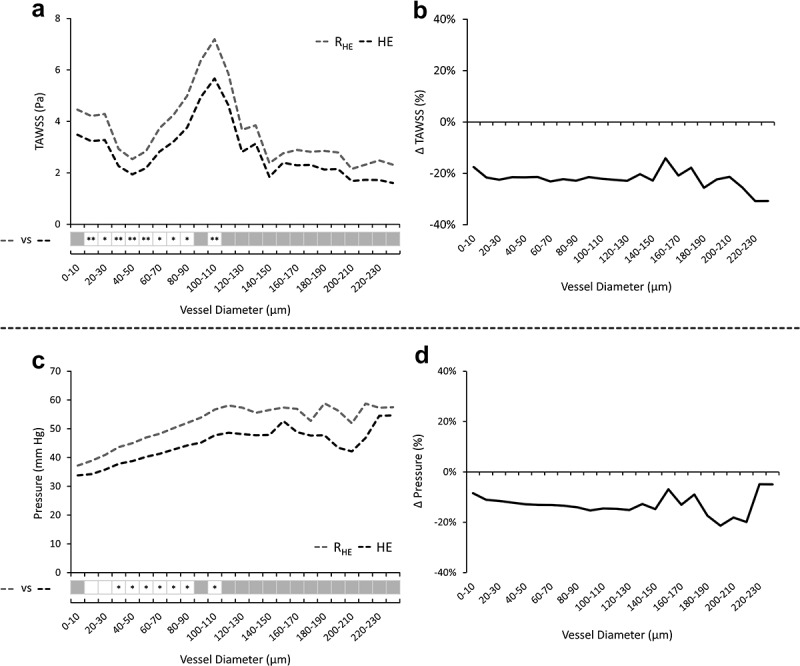
Averaged hemodynamic metrics discretized into bands by vessel diameter. Time-averaged wall shear stress (TAWSS) (a) and lumen wall pressure (c) are depicted at rest (R_HE_) and in response to heated exercise (HE). A corresponding matrix of hypothesis testing between vessel banded metrics within comparable bands (white squares) is displayed below each hemodynamic metric plot. Stars (*) indicate the level of significance (*p < 0.05; **p < 0.001) using paired *t*-tests. Vessel banded relative changes in TAWSS (b) and pressure (d) are also shown.

Hemodynamic metrics averaged across vessel diameter bands at rest and following HE, along with the corresponding vessel banded relative changes are presented in Figure 7. Across all comparable diameter bands, paired *t*-testing indicated TAWSS was significantly lower in response to HE (*p* range 0.001-0.002), with a mostly uniform average relative change of −22 ± 4%. Outside of 10–30 µm vessels, lumen pressure was also significantly lower in response to HE across remaining comparable bands (*p* range 0.006–0.039), with a similarly uniform lower average relative change of −13 ± 4% in response to HE compared to R_HE_.

## Discussion

The rationale behind this study was to assess the impact of commonly experienced and ecologically valid environmental heating and exercise exposures on distinct regions and levels of the arterial tree, using novel and quantitative micro- and macrovascular techniques. In this study, we evaluated extracranial, skin and retinal responses to environmental PH and HE stimuli. Physiological assessments were made using duplex ultrasound and OCT-based methods, while retinal arteriolar hemodynamics were assessed using subject-specific eye imaging and 3D CFD simulation. Our findings indicate that PH increased indices of skin blood flow, but decreased retinal flow. Exercise during time matched environmental heat exposure exacerbated these differences. These findings suggest that hemodynamic changes are artery specific, with implications for eye and brain responses to ecologically challenging physiological stimuli.

Our finding that PH and HE increase skin blood flows is based on responses observed using OCTA on the forearm, and also that increases in CCA blood flow are a surrogate for changes in the external carotid artery (ECA). In response to PH, and particularly HE, CCA flows generally increased, in the absence of large changes in ICA flows. This suggests that ICA flows to the brain were autoregulated in response to both stimuli, and that the relatively larger CCA changes were likely due to changes in the ECA subserving skin vasodilation in the scalp and face. Interestingly, retinal flows decreased in response to both PH and HE. The observation of stable responses in the ICA despite significant decreases in CRA flows suggests that intracranial distribution of blood flow may not be uniform in response to PH or HE, although further work is required to confirm this. Our observation that skin blood flow change occurs in an opposite direction to changes in the retina also suggests that microvascular responses to physiological stimuli are not universally similar across all microvascular beds.

Previous studies of the impact of PH on cerebral blood flows have reported similar findings to ours, although no previous study has completed the comprehensive set of measures we collected. In response to whole-body heating using a perfusion suit whilst supine, Ogoh *et* *al.* showed that, at similar increases in T_C_ to our study, ICA flow did not change, while ECA flow increased [40]. Similar findings have been reported by others [41–43]. Larger increases in T_C_ can induce larger increases in the ECA flow, and possibly, decreases in ICA flow [40,44]. In another study, Caldwell *et al*. found that regional cerebrovascular differences may exist in response to mild-to-moderate perfusion-based heating, as ICA flow remained unchanged, whereas vertebral artery (VA) flow increased [16]. Taken together, these studies indicate that, whilst global intracranial blood flow responses to PH remain largely autoregulated, particularly compared to the large changes that occur in cardiac output and skin blood flows, there may nonetheless be differences in blood flow distribution within the brain, a finding consistent with the distinct changes we observed between the CRA and ICA in the current study.

The addition of exercise to a PH load challenges systemic cardiovascular homeostasis [1,5,6], as vasodilator capacity of the skin and exercising muscle theoretically exceeds the ability of cardiac output to maintain blood pressure. However, no previous studies, to our knowledge, have compared responses to heat and/or exercise between retinal, skin and cerebrovascular feed arteries. A review of exercise effects on intracranial blood flows concluded a biphasic response exists in the ICA, with initial increases in blood flow as exercise intensity increases, which then plateaus at moderate-to-high intensity [45]. Broadly in keeping with this, Sato *et* *al.* found that short duration (~5 min) moderate (40% and 60% VO_2_max) intensity recumbent cycling significantly elevated ICA flow, which returned to baseline at higher intensities (80% VO_2_max) [46]. In a separate study, Sato *et* *al.* also assessed the impact of adding exercise whilst undergoing environmental heating, and observed that ICA and VA flows were lower relative to the combined exercise and heat conditions, whilst CCA flow was enhanced by the addition of exercise to heat [47]. Recent work by Gibbons *et al*. also observed ICA and VA blood flows that returned toward baseline conditions under similar environmental heating, exercise conditions and T_C_ rise (40% VO_2_peak, 38°C in non-breathable clothing, T_C_ +1.5°C) [2]. These findings are consistent with the results in the current study, in that ICA flows were similar to baseline levels in response to HE, whereas CCA flow increased significantly. However, retinal and skin microvascular blood flow and/or 3D hemodynamics were not recorded or compared in these studies.

Some previous studies have assessed retinal vascular relative changes during heat and/or exercise exposures. In response to passive body heating, Ikemura *et al*. observed decreased retinal BF, measured using laser-speckle imaging [48], a finding in keeping with our CRA ultrasound results in response to PH. Cycling to exhaustion under thermoneutral conditions (20°C) resulted in initial increases in retinal BF from baseline, which returned toward baseline levels as exercise continued [49]. These investigators also observed that the addition of exercise to a heat load decreased retinal flows below baseline levels as the exposure continued [49]. While these studies were not designed to compare macro- and microvascular effects of heat and exercise, or differences between microvascular territories, our results comparing the impacts of PH and HE on contemporaneous measures of conduit and microvascular responses are generally consistent with previous studies.

The extracranial blood flow measures we assessed in the current study incorporated direct and continuous edge-detection and wall tracking of B-mode diameters, in concert with Doppler velocity assessment, under PH and HE conditions. Whilst Doppler ultrasound measures of CRA velocity were also collected, CRA *flow* assessment was based on the assumption of constant diameter in our study, which is a limitation. Interestingly, Jensen *et al*. found that PH (T_C_ +1.1°C vs. the PH + 0.2°C and HE + 1.4°C T_C_ changes in this study) resulted in an increase in retinal artery diameter (+2.8%), measured using CRAE estimation from pre and post heating fundus images [50]. Similar increases (+1.7%) in CRAE during submaximal treadmill exercise (40 mins) under thermoneutral conditions have also been observed [51]. If we assume dilations similar to these studies occurred in response to PH or HE in the current experiment, our CRA flow estimates nonetheless remain directionally different to those observed in the ICA (i.e. a decrease rather than increase; see Supplementary Figure 11, which explores the effect of CRA diameter variation on calculated CRA BF responses to PH and HE). It is also germane that our estimates of WSS, a stimulus for endothelium-mediated artery dilation, decreased in our experiment in response to HE and this trend in WSS would remain regardless of the CRA diameter assumptions adopted. We are therefore confident that our study similarly demonstrates differential changes between the ICA (stable or small increase) and CRA (decrease) in response to environmental heating and exercise stimuli.

In addition to the vascular outcomes considered above, we also simulated and calculated 3D retinal microvascular hemodynamics, which we compared to OCT-derived skin microvascular responses. Following HE, we observed a significant interaction, indicating that skin blood flow measured using OCTA increased, whilst retinal vessel flows decreased. This finding reinforces our discussion above, regarding distinct changes in different microvascular territories in response to HE. It further reflects the notion that increased cardiac output in response to HE is distributed toward the skin to subserve thermoregulatory heat loss, in preference to the ICA or retinal vessels.

From the simulations we calculated TAWSS distributions that were stratified by vessel diameter. This revealed a pattern characterized by a trough in TAWSS at arteries ~40-50 μm, and a peak at ~100-120 μm in diameter. This reflects a similar distribution found by Rebhan *et al*. in a previous healthy eye simulation [38]. The 40–50 μm trough may be explained by a larger proportion of vessel diameters residing within this band, which may be associated with the bifurcating vessels leading from the CRA into the retinal superficial plexus. These additional vessels may provide parallel flow pathways, ultimately decreasing the relative flow across arterioles of this caliber. Coupled with a minima in the blood viscosity model we implemented (where viscosity is proportional to WSS), this results in a decrease in WSS. Conversely, the peak in WSS at 100–120 μm may be explained by a reduction in the proportion of branching vessels, which reduces the parallel flow networks available to distribute the relatively higher flow entering from the CRA. This ultimately increases flow relative to vessel caliber and therefore WSS. In response to HE, uniform decreases in TAWSS and lumen pressure were found across all vessel bands, despite the fact that we implemented non-linear outlet boundary conditions (i.e. Windkessel models). This uniformity is consistent with previous simulations that used simpler outlet boundary conditions [39]. Our results suggest that any systemic or induced upstream changes in flow to the eye may result in uniform changes in 3D hemodynamics throughout the retinal vasculature.

While this study provides insights into cerebral, retinal and skin hemodynamic responses to environmental heat and exercise, it possesses limitations and creates avenues for future work. We recruited a relatively small cohort of participants and, whilst statistical significance was observed, future studies would ideally increase sample size. While a number of key contemporaneous physiological measures were collected pre and post environmental passive heating and heated exercise, additional systemic or peripheral measures such as stroke volume and cardiac output, other cerebral artery responses (e.g. VA and/or middle cerebral artery velocities) or peripheral artery responses (e.g. brachial artery flow) [52,53], may shed further light on regional differences in arterial adaptation in future studies. Furthermore, collection of cardiorespiratory measures reflective of arterial CO_2_, such as end-tidal partial pressure of CO_2_, may provide further insight into the mechanisms driving cerebrovascular and retinal blood flow changes in response to thermal stimuli. While this study measured skin blood flow via OCTA methods using a system designed to assess forearm skin blood flow, future work may benefit from evaluating skin microvascular responses in areas supplied by the extracranial arteries, such as at the forehead.

In this study, we sought to understand changes in different vascular territories under conditions that are commonly experienced by humans in day-to-day life; heat exposure and moderate exercise during hot conditions. In keeping with this ecologically valid approach, we matched time exposure rather than levels of T_C_ rise. A future experiment in which changes in T_C_ were matched between PH and HE conditions would provide further insight regarding the responses of different vascular (i.e. CCA vs. ICA vs. CRA) and microvascular beds (i.e. skin vs. retinal) in response to environmental versus combined endogenous heat exposure. Other future studies may address whether the differences we observed in blood flow changes in distinct vascular levels and regions in response to PH or HE reflect systemically or locally driven mechanisms. Methods such as four-dimensional (4D) flow magnetic resonance imaging (MRI) to observe and capture the entire distribution of flow through different vascular regions of the brain may be possible during passive heating, if not exercise in the heat. These could be further enhanced by the type of CFD simulation methods we have demonstrated in this, and previous, experiments [22,23], to ascertain network-wide hemodynamic changes (i.e. shear stress).

The 3D eye models we developed in this study used accessible techniques and equipment, however additional measures of eye asymmetry using MRI [54], or direct imaging of CRA vessel geometry using cone-beam computed tomography angiography [55], may improve these models. Additional imaging to collect retinal flow data using retinal Doppler OCTA may also provide simulation validation. Finally, while our fluid simulations incorporated some of the effects of downstream vessel resistance and compliance provided by the capillaries and the retinal venules (i.e. Windkessel modeling with minimum pressure limited to IOP measurement, as an indicator of retinal venous pressure), future work could extend these methods to account for changes in venous reactivity [56,57] or autoregulation [58]. Furthermore, within the simulated arterial domains we assumed rigid wall vessels, rather than allowing them to deform using methods such as fluid structure interaction (FSI). However, previous retinal simulations found relatively uniform decreases in WSS between FSI and rigid wall simulation [38]. As such, while absolute TAWSS may be higher in our simulations, the relative change comparisons in response to HE from rest may minimize this variability.

In summary, we evaluated skin, extracranial and retinal vascular responses to environmental PH and HE, using physiological assessments and simulations. Our data indicated that while PH increased indices of skin blood flow, retinal flow decreased despite relatively stable ICA flow. The addition of exercise in the heat under duration-matched environmental conditions significantly exacerbated these differences. These findings further indicate that in response to environmental heat and exercise, distinct conduit and microvascular territories respond in different ways, and that flow may also be redistributed idiosyncratically within the brain. These findings may have implications for cerebral and retinal hemodynamic changes that occur during exposure to ecologically relevant thermoregulatory challenges.

## Supplementary Material

Revised - Supplementary Information.docx

## Data Availability

The data generated during and/or analyzed during the current study are included in this article, and within the supplementary information. Simulation files are not publicly available due to file size limitations but are available from the corresponding author upon reasonable request.

## Abbreviations

3D: Three-dimensional
4D: Four-dimensional
BF: Blood flow
BFv: Blood flow velocity
BMI: Body mass index
CAD: Computer aided design
CCA: Common carotid artery
CFD: Computational fluid dynamics
CRA: Central retinal artery
CRAE: Central retinal artery equivalent
ECA: External carotid artery
FSI: Fluid structure interaction
HE: Heated exercise
HR: Heart rate
ICA: Internal carotid artery
IOP: Intraocular pressure
LDF: Laser Doppler flowmetry
MRI: Magnetic resonance imaging
O_2_: Oxygen
OCT: Optical coherence tomography
OCTA: Optical coherence tomography angiography
OPP: Ocular perfusion pressure
PeCO_2_: Mixed expired partial pressure of carbon dioxide
PH: Passive heating
RH: Relative humidity
R_HE_: Rest prior to heated exercise
R_PH_: Rest prior to passive heating
TAWSS: Time-averaged wall shear stress
T_C_: Core temperature
UWF: Ultra-widefield fundus
VA: Vertebral artery
VO_2_: Rate of oxygen consumption
VO_2_max: Maximal rate of oxygen consumption
VO_2_peak: Peak rate of oxygen consumption
WBT: Wet bulb temperature
WSS: Wall shear stress
