# Supplementary material for: Retinal and cerebral hemodynamics redistribute to favor thermoregulation in response to passive environmental heating and heated exercise in humans
Source: Temperature (Austin). 2024 Oct 16;12(1):55–70. doi: 10.1080/23328940.2024.2411771 (PMC11875494; doi:10.1080/23328940.2024.2411771)
Supplement: Revised - Supplementary Information.docx [file KTMP_A_2411771_SM8384.docx]

Supplementary Information for “Retinal and cerebral hemodynamics in response to environmental heat and exercise in humans”

Harrison T. Caddy^1,2^, Jesse L. Criddle^2^, Kristanti W. Wigati^2,3^, Howard H. Carter^2^, Lachlan J. Kelsey^1,4^, Alla Soloshenko^5^, William H. Morgan^5,6,7^, Barry J. Doyle^1,4^, Daniel J. Green ^2^

^1^Vascular Engineering Laboratory, Harry Perkins Institute of Medical Research, Queen Elizabeth II Medical Centre, Nedlands, Australia and the UWA Centre for Medical Research, The University of Western Australia, Perth, Australia

^2^School of Human Sciences (Exercise and Sport Sciences), The University of Western Australia, Perth, Australia

^3^Medical Physiology and Biochemistry Department, Faculty of Medicine, Universitas Airlangga, Surabaya, Indonesia

^4^School of Engineering, The University of Western Australia, Perth, Australia

^5^Lions Eye Institute, Perth, Australia

^6^Centre for Ophthalmology and Visual Science, The University of Western Australia, Perth, Australia

^7^International Space Centre, Perth, Australia

**Short Title:** Hemodynamic responses to environmental heat and exercise

**Corresponding Author:** Daniel J. Green, The University of Western Australia M408, Crawley WA 6009, Australia. Phone +61-8-6488 2361. E-mail: [danny.green@uwa.edu.au](mailto:danny.green@uwa.edu.au)

# Structural Eye Imaging

To derive 3D localized hemodynamics within participant retinal vessels (e.g., wall shear stress, pressure, microvascular outlet flows), structural imaging of the left eye and 3D model construction was performed. Specifically; Scheimpflug, ocular ultrasound, retinal optical coherence tomography (OCT) and (UWF) ultra-widefield fundus imaging data were collected for each participant, which are detailed below.

*Scheimpflug imaging*

The corneal thickness and anterior segment of the eye was imaged non-invasively using a Pentacam system (OCULUS, Wetzlar, Germany), which captured 24 Scheimpflug images of the anterior eye. Within the accompanying software, we extracted corneal diameter, as well as thicknesses from the center to obtain a corneal minimum thickness, as well as at 14 locations about the periphery of the cornea to obtain an average cornea edge thickness. Anterior chamber depth (distance from cornea to lens) [1] was also extracted.

*Ultrasound imaging*

The anterior to posterior (AP) eye length, as well as lens thickness, was imaged using a linear transducer (15L4) and portable ultrasound system (uSmart 3300, Terason, Burlington, United States) as described previously [2]. The average of a transverse and horizontal scan was used to estimate AP length and lens thickness.

*Retinal imaging*

Average retinal layer thickness measurements were estimated from OCT scans, which were captured using a Spectralis HRA+OCT system (Heidelberg Engineering, Franklin, United States). Scans were centered at the fovea and used a scan angle of 30° (pattern size ~9.0 x 7.5 mm^2^), capturing 121 B-scans (distance spacing ~63µm) using Automatic Real Time-function (ART) averaging (across ~10 images). These scans were subsequently analyzed using the explorer and segmentation tools within the Iowa Reference Algorithms (Retinal Image Analysis Lab v3.8.0, Iowa Institute for Biomedical Imaging, Iowa City, IA) [3-6]. The mean thickness from thickness maps of the retinal nerve fiber layer (NFL), ganglion cell and inner plexiform layers (GCL+IPL), inner nuclear layer (INL), outer plexiform layer (OPL), outer nuclear layer and photoreceptor inner segment (ONL + PIS) and photoreceptor outer segment (POS) were extracted for each participant.

Average choroid thickness was estimated from OCT enhanced depth imaging (EDI) scans, captured using the same Spectralis HRA+OCT system. These scans were again centered at the fovea with an angle of 30°, consisting of 121 B-scans using ART averaging. Imaging data was exported and subsequently cropped in Fiji/ImageJ (v1.53t), prior to being analysed using open-source choroid thickness mapping software as described by Mazzaferri *et al.* [7] in MATLAB (v2019b, Mathworks, Natick, United States). The resulting choroid thickness map was then manually thresholded to omit erroneous instantaneous increases in thickness (due to scan signal drop out), then averaged to obtain a mean choroid thickness.

An additional 30° OCT circumpapillary scan using the same Spectralis HRA+OCT system was also captured and the fundus representation extracted to estimate the diameter of the central retinal artery. For this, we used the revised equations [8] of the Parr-Hubbard formula for calculation of the central retinal artery equivalent (CRAE) [9-11]. This process involved combining the diameters of the six largest arterioles within a 0.5-1 optic disc diameter sized zone to estimate a parent branch diameter indicative of the central retinal artery. To quantify vessel diameter, we imported the OCT extracted fundus image into the Automated Retinal Image Analyser (ARIA) [12], which runs within MATLAB (v2019b). Within ARIA, we used the profile plot to omit vessel diameter artefacts. For a test case, we found that CRAE estimated from either manual segmentation within the accompanying Heidelberg software system, or the semi-automated segmentation approach with ARIA, were within 5% of each other. The central retinal venule equivalent (CRVE) was also extracted using this same method to enable calculation of the retinal arteriovenous ratio (AVR), defined as CRAE/CRVE.

The superficial retinal plexus was captured using ultra-widefield (UWF) fundus imaging (Optos P200DTx, Optos, Dunfermline, Scotland). The optic disc diameter was measured within the OptosAdvance (Optos) cloud-based image management system.

*Parametric 3D eye model*

Parametric computer aided design (CAD) representations of the eye (based on past studies [13-16]) were generated in Fusion360 (v16.3.0.2035, Autodesk, San Francisco, United States) which incorporated measurements from structural eye imaging discussed above to obtain estimates for the retinal plexus spherical curvature radius and estimates of the CRA diameter using the central retinal artery equivalent (CRAE) [8-11], as depicted in Supplementary Figure 1.

**Supplementary Figure 1.** Parametric computer aided design (CAD) representation of the eye, including example images of eye imaging data (**A**). Corneal diameter (**i**), corneal center (**ii**) and edge (**iii**) thicknesses as well as anterior chamber depth (**iv**) were measured from Scheimpflug images. Note, the sclera thickness was assumed to be equal to corneal edge thickness. Ultrasound imaging was used to estimate lens thickness (**v**) and anterior-posterior length to the start of the retina (**vi**). The various layers of the retina and the choroid (**vii**) were estimated from fovea centered optical coherence tomography (OCT) imaging. Acting out of plane (not depicted) is the optic nerve sheath assumed to be the same diameter as the optic disc, which was measured from ultra-widefield fundus imaging. Following input of imaging measurements into the parametric CAD template, different eye components were generated (**B**), enabling the construction of a whole eye model (**C**) and estimation of the retinal plexus curvature radius and central retinal artery equivalent (CRAE) diameter used in subsequent retinal plexus 3D model generation and fluid simulation.

*3D superficial retinal plexus*

The 3D models of the superficial retinal vascular plexus required for computational fluid dynamics (CFD) simulation were generated as depicted in Supplementary Figure 2. Initially, high resolution fundus images of the superficial retinal plexus captured using UWF imaging (Optos P200DTx, Optos, Dunfermline, Scotland) were exported from OtposAdvance (Optos), which were manually adjusted (i.e., brightness, contrast and gamma via green channel) to improve image clarity and consistency across cases (Supplementary Figure 2A). Exported images were processed in Fiji/ImageJ (v1.53t) to enhance contrast further using the Contrast Limited Adaptive Histogram Equalization (CLAHE) algorithm, before importing the image into MATLAB (v2019b). In MATLAB, we used a series of homomorphic and Frangi filtering techniques, as described previously [17], to generate thresholded binary images of the retinal vasculature (Supplementary Figure 2B). These binary images were then segmented to obtain the retinal arterioles (identified by a generally smaller diameter and color differences (i.e., brighter) [18]) and manually cleaned of thresholding artifacts in the GNU Image Manipulation Program (GIMP, v2.10.36) (Supplementary Figure 2C). Binarized retinal arteriole images were then imported back into MATLAB for skeletonization to obtain centerline and diameter information using custom scripts (Supplementary Figure 2D). This data was then projected to the spherical curvature of the retina obtained from the parametric CAD eye representations (Supplementary Figure 2E). Retinal plexus vessels were assumed to remain within the middle of the nerve fiber, ganglion cell and inner plexiform layers [19]. Additionally, we included a vessel that was projected outwards from the optic disc to represent the CRA, with an assumed diameter equal to the previously calculated CRAE and an assumed length of 5 mm [20]. Finally, this spherically projected vessel centerline and diameter information was then used in CAD operations within STAR-CCM+ (v18.02.010, Siemens, Munich, Germany) to generate lofted tubular structures representative of the retinal arteriole vasculature, which were smoothed at bifurcation points (Supplementary Figure 2F).


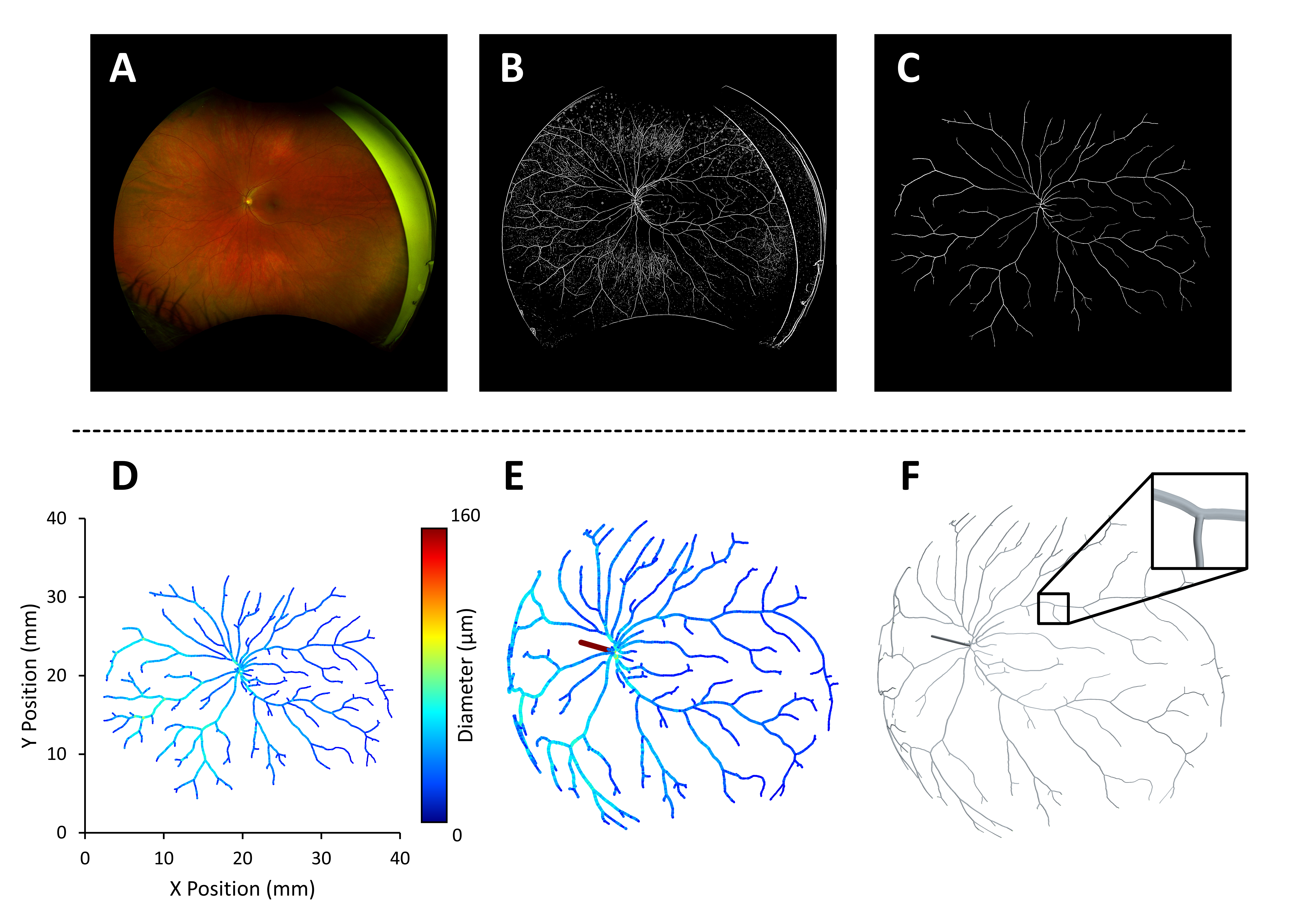


**Supplementary Figure 2.** Process for generation of 3D superficial retinal arteriole vascular plexus for an example case. Ultra-widefield fundus images were exported (**A**) and processed using Frangi filtering and binary thersholding in MATLAB (**B**), before manual cleanup of thresholding artefacts and identification/seperation of the arteriole network in GIMP (**C**). Binarized images were imported into a custom MATLAB script to calculate centerline skeleton and diameter data (**D**), before being projected to the curvature of a 3D sphere (**E**). This centerline and diameter information was then imported into STAR-CCM+ and lofted tubes were created using computer aided design (CAD) tools (**F**), which were smoothed at bifurcations (detail view) using surface repair tools.

# CRA Flow Waveforms


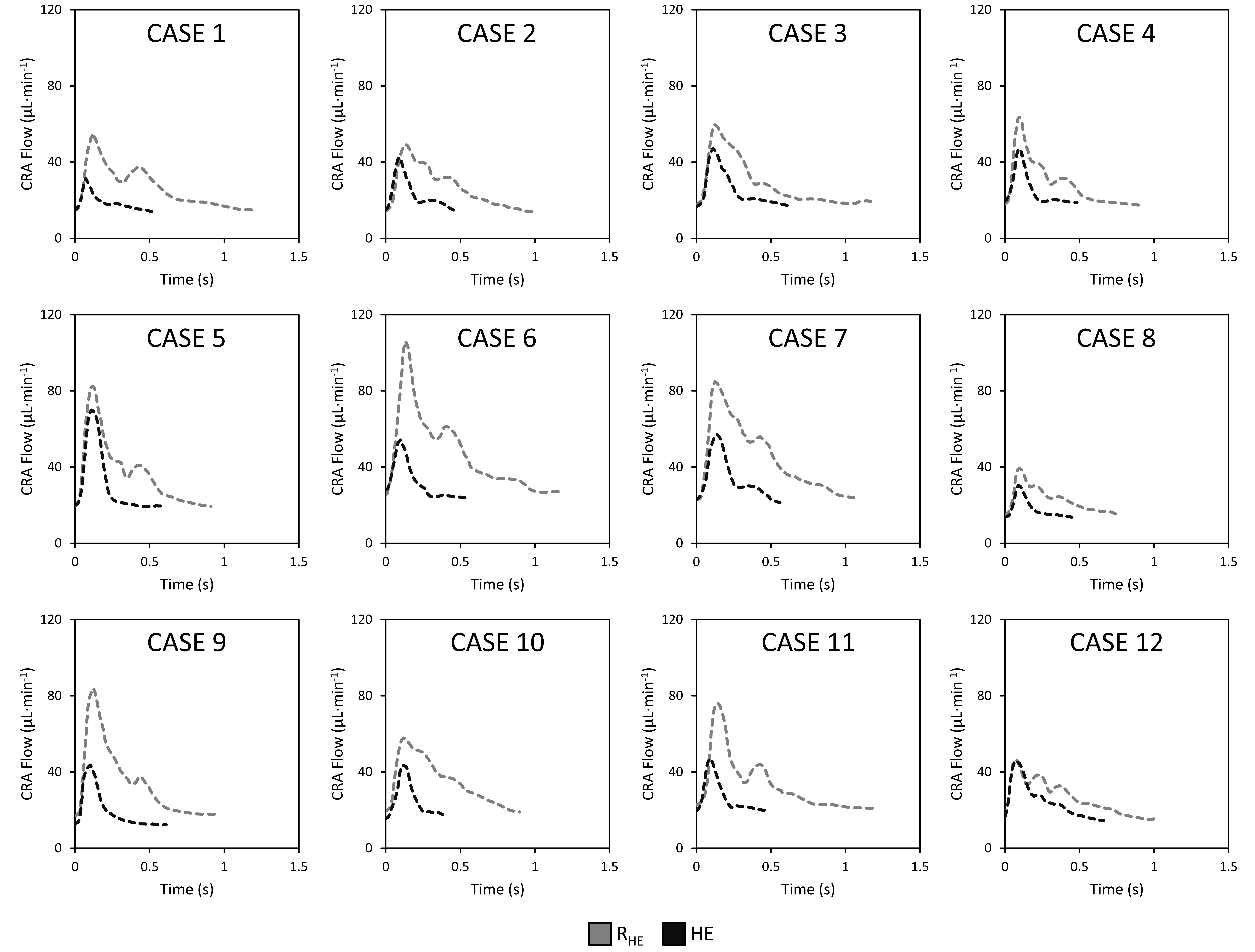


**Supplementary Figure 3.** Central retinal artery (CRA) flow waveforms for each case at rest (R_HE_) and then following heated exercise (HE) for all cases. These waveforms were calculated by combining the blood flow velocity from Doppler ultrasound with the diameter estimated from the central retinal artery equivalent (CRAE) calculated from a fundus image extracted from optical coherence tomography (OCT) data.

# Computational Fluid Dynamics Simulation

*Computational Mesh*

To generate the computational mesh we used a combination of polyhedral mesh elements within the core of the lumen and anisotropic prism layer elements for near wall refinement. In order to ensure mesh independence, we used the non-uniform refinement ratio formulation of the grid convergence index (GCI) [21,22] with the case with the highest average inflow, as well as highest Reynolds and Womersley numbers. Hemodynamic metrics of interest remained below 2%, indicating sufficient discretization – in line with past retinal simulations [17,23]. GCI sweeping parameters for meshing (Supplementary Table 1), final mesh settings applied for each subsequent case (Supplementary Table 2), and GCI results (Supplementary Table 3) for hemodynamic metrics of interest are presented below. For conservatism, the inlet flow waveform for the selected case was scaled to double the amplitude. Across all cases, final meshes consisted of ~28 ± 6 million elements.

**Supplementary Table 1.** Sweeping mesh parameters for generating coarse, medium and fine meshes for GCI analysis for the case with the highest Womersley and Reynolds numbers.

| **Meshing Parameter** | **Coarse Mesh** | **Medium Mesh** | **Fine Mesh** |
| --- | --- | --- | --- |
| Number of Prism Layers | 3 | 6 | 12 |
| Prism Layer Stretching Ratio | 1.414 | 1.149 | 1.065 |
| Volume Growth Rate | 1.8 | 1.4 | 1.2 |
| Number of Elements | 9,146,110 | 14,363,743 | 24,786,500 |

**Supplementary Table 2.** Final surface and volume mesh settings and parameters used across each case.

| **Meshing Model** | **Meshing Parameter** | **Value** |
| --- | --- | --- |
| Surface Remesher | Target Surface Size (µm) | 10 |
|  | Minimum Surface Size (µm) | 1 |
|  | Surface Curvature (points/circle) | 40 |
|  | Surface Growth Rate | 1.3 |
| Polyhedral Volume Meshing | Prism Layer Number | 12 |
|  | Prism Layer Stretching | 1.065 |
|  | Prism Layer Thickness (µm) | 15 |
|  | Volume Growth Rate | 1.2 |

**Supplementary Table 3.** GCI results for hemodynamic metrics of interest. Results for surface averages of time-averaged wall shear stress (WSS) and wall pressure are presented.

| **CFD Metric** | **GCI** |
| --- | --- |
| TAWSS (Pa) | 0.09% |
| Pressure (mm Hg) | 0.10% |

*Blood and Domain Properties*

We assumed a laminar flow regime given, across all cases, the maximum Reynolds number for inlet flow (*Re*= 6.4) was orders of magnitude below values associated with transition to turbulence [24], in addition to low Womersley numbers across all cases (all < 0.17) [25]. Blood was assumed to be incompressible with a density of 1050 kg·m^-3^ [17,26], and we implemented a viscosity model to account for the Fåhræus–Lindqvist effect, as described previously by Pries *et al.* [27] assuming a hematocrit of 0.45 for all cases.

We accounted for the effects of gravity, assuming an acceleration of 9.81 m·s^-2^ [28] which acted inferiorly across all cases and conditions, representative of the upright seated posture maintained throughout testing.

Initial domain arterial pressure (P_a_) was assumed to be equal to 7/10 MAP – 19.5 mm Hg [29], where MAP is mean arterial pressure.

*Boundary Conditions*

Lumen walls were assumed to be rigid and used a no-slip wall boundary condition [17].

For each case and condition (i.e., rest; R_HE_ and heated exercise; HE) we prescribed time varying flow waveforms at the central retinal artery (CRA) inlet surface calculated from the Doppler blood flow velocity (BFv) waveform measured in the CRA using ultrasound and central retinal artery equivalent (CRAE) diameter.

At each of the outlet boundaries we prescribed pressure outlets which used 3-element Windkessel models combined with a calculated fractal tree resistance. Specifically, we used the distal resistance (6·10^15^ Pa·s·m^-3^) and compliance (1.67·10^-14^ m^3^·Pa^-1^·s^-1^) constants previously described for 3-element Windkessel modelling in retinal arterioles [30]. We then set the proximal element to a resistance calculated using structured asymmetrical fractal trees as per previously described methods [23,31,32]. The minimum outlet pressure was limited to the correspondingly measured IOP [33].

*Simulation Execution*

We used the unsteady segregated flow solver using the semi-implicit method for pressure-linked equations (SIMPLE) algorithm with 2^nd^-order temporal discretization. Simulations ran for 2 cardiac cycles, with 1000 time steps per cardiac cycle [34] and 20 inner iterations per time step, which terminated earlier if normalized momentum and continuity residuals fell by 10^-4^. Time averaged data was collected over the second cardiac cycle, which was sufficient for variable stabilization. Simulations were executed using Setonix, an HPE Cray EX supercomputer (Pawsey Supercomputing Centre, Perth, Australia). Simulations were run across 10 nodes utilizing a collective of 1280 cores, requiring approximately 11 ± 3 h of wall time (~14,000 core hours) per case and condition.

# Cardiorespiratory and Temperature Metrics


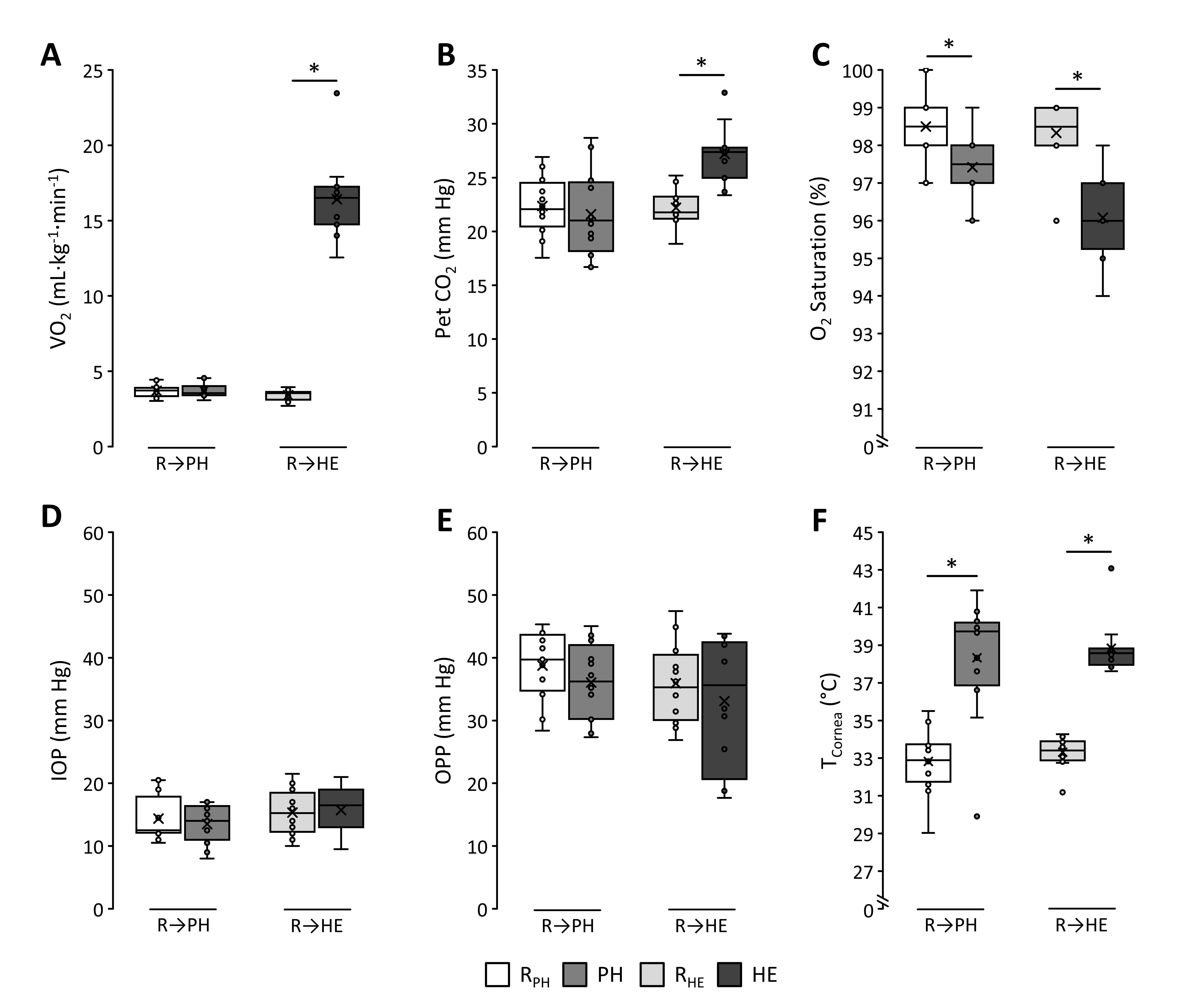


**Supplementary Figure 4.** Additional cardiorespiratory and temperature metrics. Volume of oxygen consumed (VO_2_) (**A**), mixed expired partial pressure of carbon dioxide (PeCO_2_) (**B**) and peripheral oxygen (O_2_) tissue saturation (**C**), intraocular pressure (IOP) (**D**), ocular perfusion pressure (OPP) (**E**), and surface average corneal temperature (T_Cornea_) (**F**) responses to passive heating (PH) from rest (R_PH_) and to heated exercise (HE) from rest (R_HE_). Stars (*) indicate the level of significance (*P < 0.05; **P < 0.001) using paired t-tests while crosses (†) indicate significant interactions (†P < 0.05; ††P < 0.001) using 2-way repeated measures ANOVA.

.

# Heat Stress Symptoms


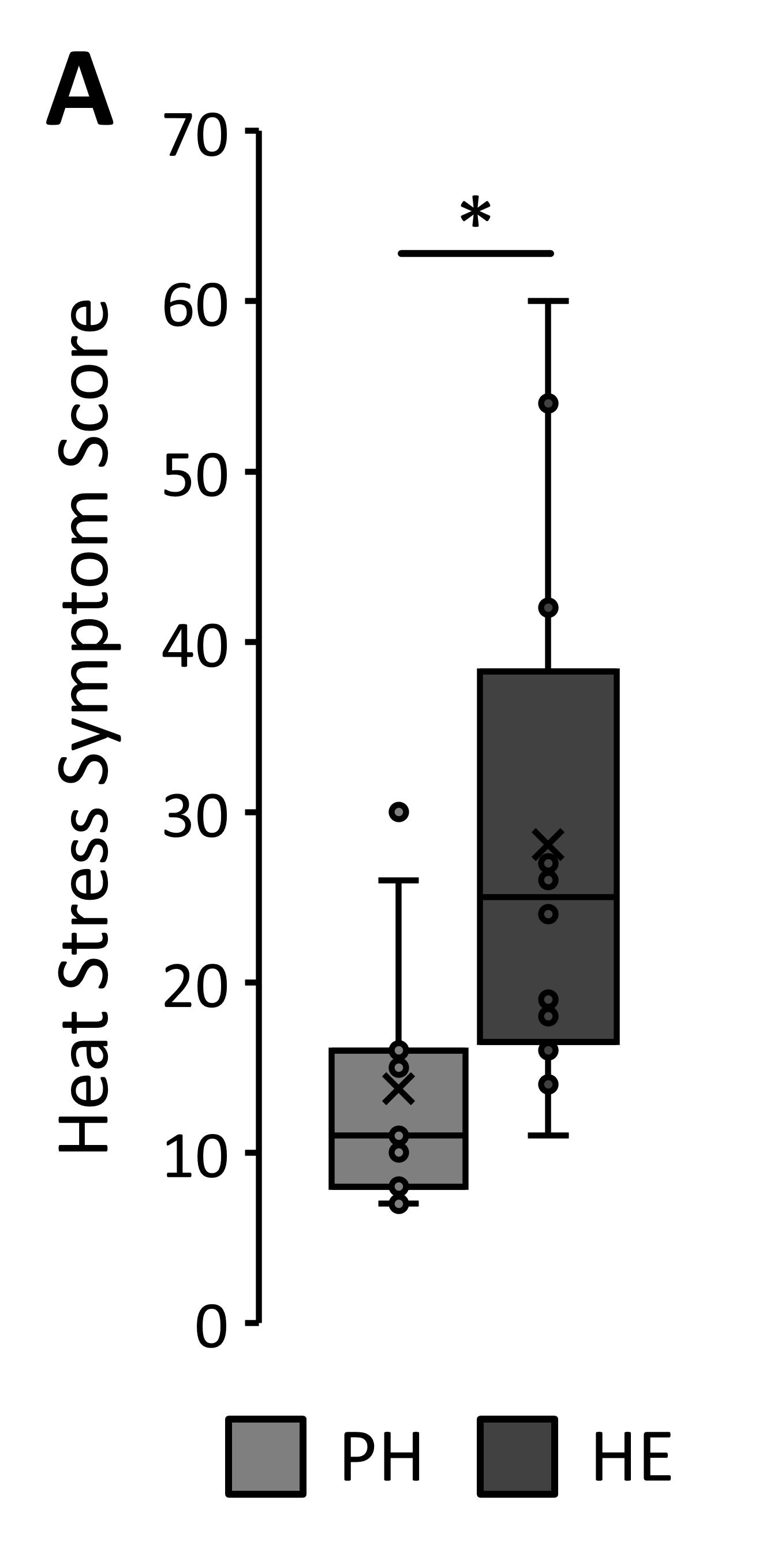


**Supplementary Figure 5.** Heat stress symptom score responses following passive heating (PH) and heated exercise (HE) (**A**). Stars (*) indicate the level of significance between conditions (*P < 0.05; **P < 0.001) using paired t-tests.

# 3D Eye Geometry Data

The parametric schematic of the CAD representation of the eye model used to generate each case is presented in Supplementary Figure 6. Grouped results for each eye geometry parameter that was imported into the CAD eye template for each case can be found in Supplementary Table 4, along with grouped mean thicknesses of each of the identified layers of the retina (Supplementary Table 5), measured from optical coherence tomography imaging (OCT). Note, total retinal thickness was calculated as the sum of these individual layers. Cohort geometry statistics from the 3D retinal arterioles utilized in CFD simulations are presented in Supplementary Table 6.


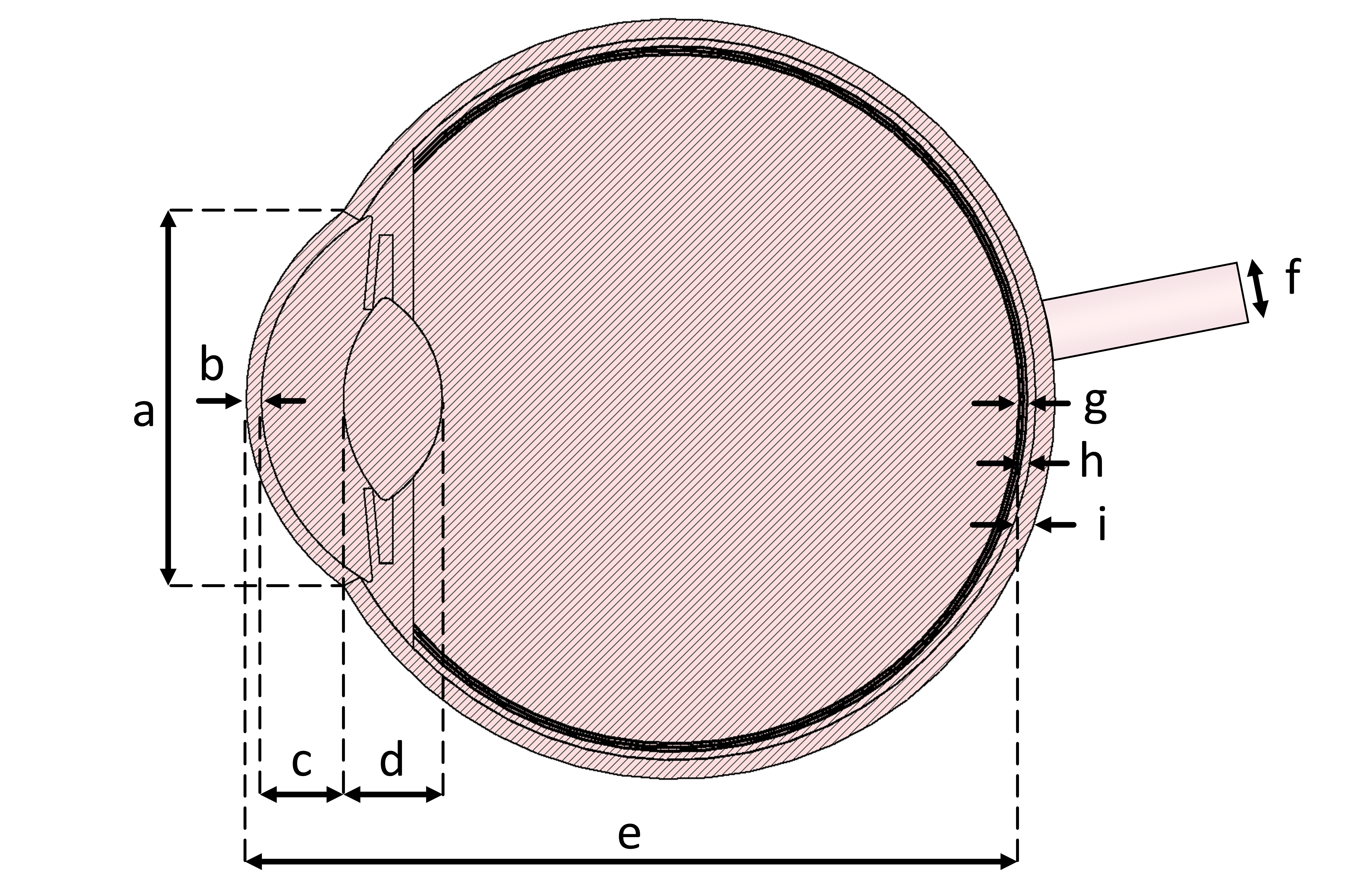


**Supplementary Figure 6.** Template representation of the eye geometry used across all cases. Measurements obtained from eye imaging modalities were extracted for each case and parametrically applied to this model to create semi-subject specific idealized eye models. The required parameters to define the geometry are cornea diameter (a), cornea thickness (b), anterior chamber depth (c), lens thickness (d), anterior-posterior length (e), optic disc diameter (f), retinal layer thicknesses (g), choroid thickness (h) and sclera thickness (i).

**Supplementary Table 4.** Average grouped measurements for different eye geometry metrics that were imported into each parametric eye template.

| **Eye Geometry** | **Mean** |  | **SD** |
| --- | --- | --- | --- |
| Cornea Diameter (mm) | 11.8 | ± | 0.4 |
| Cornea Thickness (μm) | 549.2 | ± | 34.5 |
| AC Depth (mm) | 3.1 | ± | 0.2 |
| Lens Thickness (mm) | 3.2 | ± | 0.2 |
| AP Length (mm) | 24.1 | ± | 1.0 |
| Optic Disc Diameter (mm) | 1.7 | ± | 0.2 |
| Total Retina Thickness (μm) | 306.5 | ± | 10.4 |
| Choroid Thickness (μm) | 244.4 | ± | 47.8 |
| Sclera Thickness (μm) | 646.3 | ± | 37.4 |

Total retinal thickness is the summed value of 6 different retinal layers thicknesses measured using optical coherence tomography (OCT). AC = anterior chamber, AP = anterior-posterior.

**Supplementary Table 5.** Average grouped thicknesses of the layers of the retina measured from optical coherence tomography (OCT) scanning at the fovea.

| **Retina Layer Thickness** | **Mean** |  | **SD** |
| --- | --- | --- | --- |
| NFL (μm) | 54.4 | ± | 6.7 |
| GCL + IPL (μm) | 61.3 | ± | 3.8 |
| INL (μm) | 29.1 | ± | 1.6 |
| OPL (μm) | 23.9 | ± | 1.5 |
| ONL + PIS (μm) | 89.3 | ± | 3.9 |
| POS (μm) | 48.4 | ± | 2.0 |

NFL = nerve fiber layer; GCL + IPL= ganglion cell and inner plexiform layers; INL = inner nuclear layer; OPL = outer plexiform layer; ONL + PIS = outer nuclear layer and photoreceptor inner segment; POS = photoreceptor outer segment.

**Supplementary Table 6.** Eye Plexus Geometry Statistics.

| **Eye Geometry Statistics** | **Mean** |  | **SD** |
| --- | --- | --- | --- |
| R_Plexus Curvature_ (mm) | 9.97 | ± | 0.47 |
| CRAE (µm) | 190.5 | ± | 18.7 |
| AVR | 0.71 | ± | 0.06 |
| D (µm) | 27.8 | ± | 2.9 |
| L (µm) | 863.5 | ± | 115.5 |
| SA (mm^2^) | 38.6 | ± | 5.1 |
| T | 1.09 | ± | 0.01 |

R_Plexus Curvature_ = spherical curvature projection radius for plexus vessels; CRAE = central retinal artery equivalent diameter, AVR = retinal arteriovenous ratio, D = average diameter of vessels within the retinal plexus, L = vessel length between plexus branch bifurcations, SA = total plexus vessel surface area, T = plexus vessel tortuosity between branch bifurcations.

# Vessel Diameter 3D Surface Maps





**Supplementary Figure 7.** Surface mapped distributions of vessel diameter for each 3D retinal arteriole plexus.

# Laser Doppler Flowmetry Results


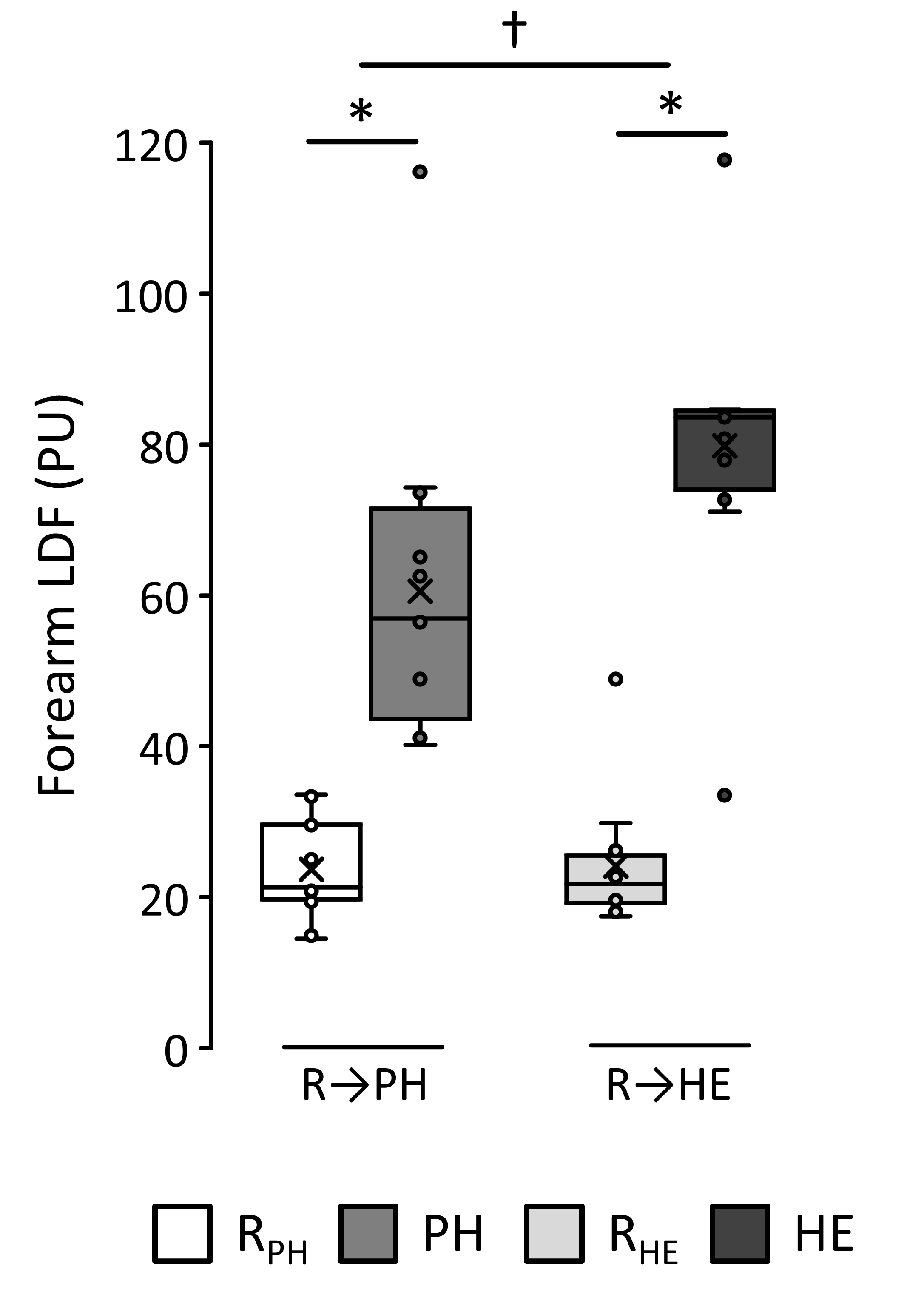


**Supplementary Figure 8.** Laser Doppler flowmetry (LDF) measurements of skin blood flux in arbitrary perfusion units (PU) in response to passive heating (PH) from rest (R_PH_) and heated exercise (HE) from rest (R_HE_). Stars (*) indicate the level of significance (*P < 0.05; **P < 0.001) using paired t-tests while crosses (†) indicate significant interactions (†P < 0.05; ††P < 0.001) using 2-way repeated measures ANOVA.

# Example Absolute Surface Distributions of Hemodynamic Metrics


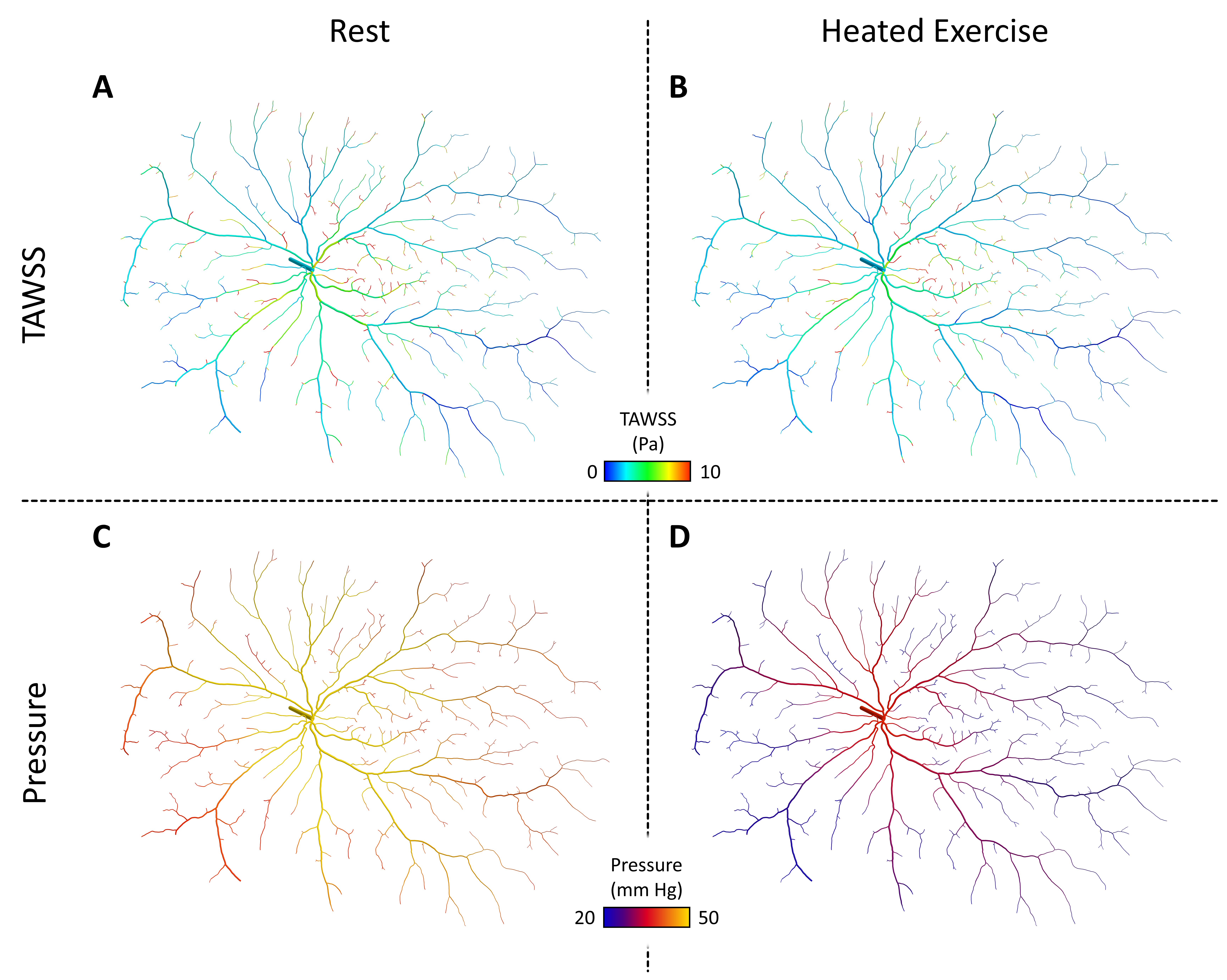


**Supplementary Figure 9.** Absolute surface distributions of hemodynamic metrics for an example case. Time-averaged wall shear stress (TAWSS) at rest (**A**) and following heated exercise (**B**), as well as wall pressure at rest (**C**) and following heated exercise (**D**) is shown.

# Relative Change in Time-Averaged Wall Shear Stress and Pressure





**Supplementary Figure 10.** Relative changes in surface distributions of time-averaged wall shear stress (TAWSS) (top) and time-averaged wall pressure (bottom) in response to HE from R_HE_ for all cases.

# Central Retinal Artery Diameter Assumption Testing


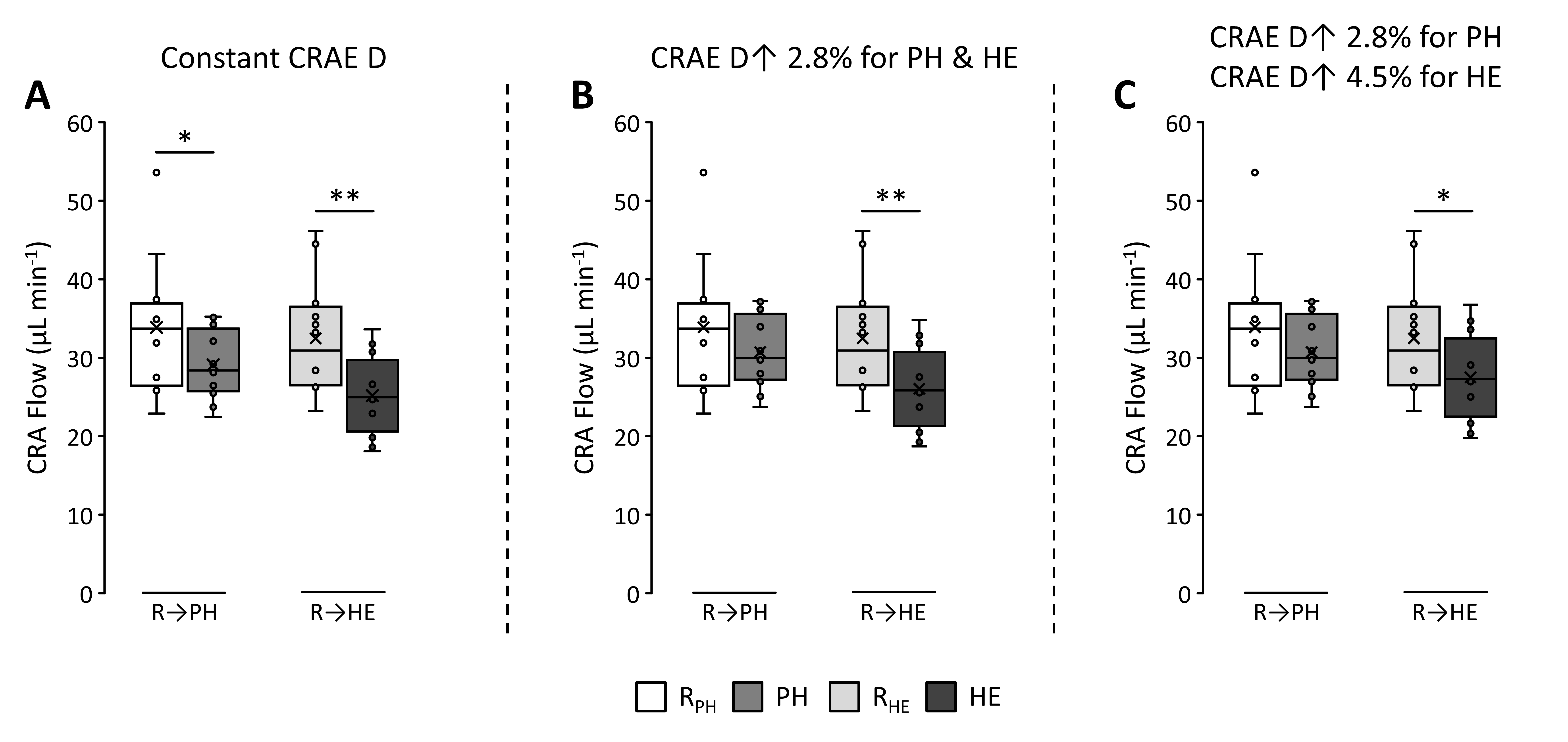


**Supplementary Figure 11.** Influence of assumed central retinal artery (CRA) diameter using the CRA equivalent (CRAE) method on CRA flow. Note, CRA flow was calculated in combination with CRA blood flow velocity measured using Doppler ultrasound. CRA flow as calculated and presented in this study with an assumed constant CRAE across both passive heat (PH) and heated exercise (HE) responses is shown (A). Distributions of CRA flow assuming a potential increase in CRAE in response to heating of 2.8% applied to both PH and HE endpoints (B), or assuming a further 1.7% dilation from the effects of exercise linearly adding with the previous dilation from heating alone for the HE endpoint (i.e., total 4.5%) (C). These additional investigations demonstrate similar trends in CRA flow across stimuli, regardless of possible assumed CRAE influence. Stars (*) indicate the level of significance (*P < 0.05; **P < 0.001) using paired t-tests.

# References

1. Cho YK, Chang HS, La TY, et al. Anterior segment parameters using Pentacam and prediction of corneal endothelial cell loss after cataract surgery. Korean J Ophthalmol. 2010;24(5):284-290.

2. Stalmans I, Vandewalle E, Anderson DR, et al. Use of colour Doppler imaging in ocular blood flow research. Acta Ophthalmol. 2011 Dec;89(8):e609-e630.

3. Abràmoff MD, Garvin MK, Sonka M. Retinal imaging and image analysis. IEEE Rev Biomed Eng. 2010;3:169-208.

4. Garvin MK, Abràmoff MD, Wu X, et al. Automated 3-D intraretinal layer segmentation of macular spectral-domain optical coherence tomography images. IEEE Trans Med Imaging. 2009;28(9):1436-1447.

5. Antony B, Abràmoff MD, Tang L, et al. Automated 3-D method for the correction of axial artifacts in spectral-domain optical coherence tomography images. Biomedical Opt Express. 2011;2(8):2403-2416.

6. Guo Z, Kwon YH, Lee K, et al. Optical Coherence Tomography Analysis Based Prediction of Humphrey 24-2 Visual Field Thresholds in Patients With Glaucoma. Invest Ophthalmol Vis Sci. 2017;58(10):3975-3985.

7. Mazzaferri J, Beaton L, Hounye G, et al. Open-source algorithm for automatic choroid segmentation of OCT volume reconstructions. Sci Rep. 2017;7:42112.

8. Knudtson MD, Lee KE, Hubbard LD, et al. Revised formulas for summarizing retinal vessel diameters. Curr Eye Res. 2003;27(3):143-149.

9. Parr JC, Spears GF. General caliber of the retinal arteries expressed as the equivalent width of the central retinal artery. Am J Ophthalmol. 1974;77(4):472-477.

10. Parr JC, Spears GF. Mathematic relationships between the width of a retinal artery and the widths of its branches. Am J Ophthalmol. 1974;77(4):478-483.

11. Hubbard LD, Brothers RJ, King WN, et al. Methods for evaluation of retinal microvascular abnormalities associated with hypertension/sclerosis in the Atherosclerosis Risk in Communities Study. Ophthalmology. 1999;106(12):2269-2280.

12. Bankhead P, Scholfield CN, McGeown JG, et al. Fast retinal vessel detection and measurement using wavelets and edge location refinement. PLoS One. 2012;7(3):e32435.

13. Mirnezami SA, Rajaei Jafarabadi M, Abrishami M. Temperature distribution simulation of the human eye exposed to laser radiation. J Lasers Med Sci. 2013;4(4):175-181.

14. Thompson KA, Bhardwaj R, Nguyen TD. Development of an Anatomically Accurate Finite Element Human Ocular Globe Model for Blast-Related Fluid-Structure Interaction Studies. US Army Research Laboratory Aberdeen Proving Ground United States; 2017.

15. Ooi EH, Ng EY. Simulation of aqueous humor hydrodynamics in human eye heat transfer. Comput Biol Med. 2008;38(2):252-262.

16. Heussner N, Holl L, Nowak T, et al. Prediction of temperature and damage in an irradiated human eye—Utilization of a detailed computer model which includes a vectorial blood stream in the choroid. . Comput Biol Med. 2014;51:35-43.

17. Rebhan J, Parker LP, Kelsey LJ, et al. A computational framework to investigate retinal haemodynamics and tissue stress. Biomech Model Mechanobiol. 2019;18(6):1745-1757.

18. Hanssen H, Streese L, Vilser W. Retinal vessel diameters and function in cardiovascular risk and disease. Prog Retin Eye Res. 2022;91:101095.

19. Campbell JP, Zhang M, Hwang TS, et al. Detailed Vascular Anatomy of the Human Retina by Projection-Resolved Optical Coherence Tomography Angiography. Sci Rep. 2017;7(1):42201.

20. Merriam JC, Casper DS. The entry point of the central retinal artery into the outer meningeal sheath of the optic nerve. Clin Anat. 2021;34(4):605-608.

21. Schwer LE. Is your mesh refined enough? Estimating discretization error using GCI. German LS-DYNA Forum; Bamberg: DYNAmore; 2008.

22. Roache P. Perspective: A Method for Uniform Reporting of Grid Refinement Studies. J Fluids Eng. 1994;116(3):405-413.

23. Caddy HT, Kelsey LJ, Parker LP, et al. Modelling large scale artery haemodynamics from the heart to the eye in response to simulated microgravity. NPJ Microgravity. 2024;10(1):7.

24. Ben-Dov G, Cohen J. Critical Reynolds number for a natural transition to turbulence in pipe flows. Phys Rev Lett. 2007;98(6):064503.

25. Ku DN. Blood Flow in Arteries. Annu Rev Fluid Mech. 1997;29(1):399-434.

26. Doyle BJ, McGloughlin TM, Kavanagh EG, et al., editors. From Detection to Rupture: A Serial Computational Fluid Dynamics Case Study of a Rapidly Expanding, Patient-Specific, Ruptured Abdominal Aortic Aneurysm. New York (NY): Springer New York; 2014.

27. Pries AR, Secomb TW, Gessner T, et al. Resistance to blood flow in microvessels in vivo. Circ Res. 1994;75(5):904-915.

28. Torok A, Gallagher M, Lasbareilles C, et al. Getting ready for Mars: How the brain perceives new simulated gravitational environments. Q J Exp Psychol. 2019;72(9):2342-2349.

29. Pappelis K, Choritz L, Jansonius NM. Microcirculatory model predicts blood flow and autoregulation range in the human retina: in vivo investigation with laser speckle flowgraphy. Am J Physiol Heart Circ Physiol. 2020;319(6):H1253-H1273.

30. Aletti M, Gerbeau J-F, Lombardi D. A simplified fluid–structure model for arterial flow. Application to retinal hemodynamics. Comput Methods Appl Mech Eng. 2016;306:77-94.

31. Malek J, Azar AT, Nasralli B, et al. Computational analysis of blood flow in the retinal arteries and veins using fundus image. Comput Math Appl. 2015;69(2):101-116.

32. Olufsen MS. Structured tree outflow condition for blood flow in larger systemic arteries. Am J Physiol. 1999;276(1):H257-H268.

33. Mursch-Edlmayr AS, Bolz M, Strohmaier C. Vascular Aspects in Glaucoma: From Pathogenesis to Therapeutic Approaches. Int J Mol Sci. 2021;22(9):4662.

34. Bappoo N, Kelsey LJ, Tongpob Y, et al. Investigating the Upstream and Downstream Hemodynamic Boundary Conditions of Healthy and Growth-Restricted Rat Feto-Placental Arterial Networks. Ann Biomed Eng. 2021;49(9):2183-2195.
